# Iron status and hemoglobin adjustment by altitude to define anemia in children aged 6 to 8 months living in Lima, Arequipa, Cusco and Puno

**DOI:** 10.17843/rpmesp.2023.404.12573

**Published:** 2023-12-18

**Authors:** Juan Pablo Aparco, Gabriela Santos-Antonio, William Bautista-Olortegui, Katherine Alvis-Chirinos, Patricia Velarde-Delgado, Paul Hinojosa-Mamani, Gilmer Solis-Sanchez, Flor Eliana Santa Cruz, Nelly Zavaleta

**Affiliations:** 1 National Center for Food, Nutrition, and Healthy Living, Instituto Nacional de Salud, (INS), Lima, Peru. National Center for Food, Nutrition, and Healthy Living Instituto Nacional de Salud (INS) Lima Peru; 2 General Directorate of Strategic Interventions in Public Health, Ministry of Health (MINSA), Lima, Peru. General Directorate of Strategic Interventions in Public Health Ministry of Health (MINSA) Lima Peru; 3 Nutritional Research Institute (IIN), Lima, Peru. Nutritional Research Institute (IIN) Lima Peru

**Keywords:** Child, Anemia, Altitude, Iron-Deficiency, Peru

## Abstract

**Objective.:**

To describe the iron status profile and to propose hemoglobin adjustment factors for altitude for children aged 6 to 8 months in Lima, Arequipa, Cusco and Puno.

**Materials and methods.:**

Cross-sectional study in children aged 6 to 8 months from four cities. We measured hemoglobin and other iron biomarkers, C-reactive protein (CRP), among others. To estimate the adjustment equation, we applied an exponential regression. We excluded children with iron deficiency (ID) and/or inflammation.

**Results.:**

The proportions of ID were higher in Puno and Arequipa, while inflammation did not exceed 19% in any of the cities. Hemoglobin showed an exponential increase at higher altitude. The adjustment equation was: 10.34249 x (1.00007 ^ Alt).

**Conclusions.:**

Children residing in Arequipa and Puno showed higher rates of ID and lower iron reserves; furthermore, the increase in hemoglobin by altitude was exponential, showing the need to adjust hemoglobin at altitude.

## INTRODUCTION

Anemia is a global public health problem and affects nearly two billion people, with children in developing countries being most vulnerable ^1^. Iron deficiency (ID), hemoglobinopathies and malaria are considered the main causes of anemia ^2^. The consequences of anemia on health and development have been previously described ^3^^,^^4^^)^ and it has effects that continue throughout life ^5^. The diagnosis of anemia is made by using cut-off points considering the age, sex and physiological state of the individual, as well as adjustment factors for smoking and altitude ^6^.

In 1945, Hurtado proposed correcting hemoglobin estimates at high altitude because this biomarker increases as an adaptive response to low oxygen pressure at altitude; he proposed an adjustment factor based on data from Peruvian adult males ^7^. In 1989, the United States Center for Disease Control (CDC) published adjustment factors for high altitude using data from children between 2 and 4 years old and residents at altitudes up to 2150 meters above sea level (masl), and they used data from Hurtado to estimate adjustment factors at higher altitudes ^8^. Then in 1994, Dirren, using data from Ecuadorian children under 5 years of age, found an adjustment factor similar to that of CDC ^9^. In 2001, the World Health Organization (WHO) adopted the altitude adjustment factors ^6^ published by CDC; however, the most current evidence shows a possible overestimation of anemia in residents at higher altitudes ^(10, 11)^, so WHO started to review the altitude adjustment and global anemia guidelines.

In Peru, Gonzales-Rengifo proposed not to adjust hemoglobin for altitude in the population of the southern region because they would be better adapted to altitude as occurs in Tibet ^12^; later, this same author developed an adjustment factor with data from the Nutritional Status Information System (SIEN) ^13^. Likewise, Bartolo *et al*. proposed an adjustment factor using the 2015 National Demographic and Health Survey (ENDES) ^14^; however, in both studies the models were only based on capillary hemoglobin without assessing the iron status of children or other factors that could elevate the hemoglobin level such as inflammation ^15^. Recently, Accinelli *et al*. with data from the2017 DHS, rekindled the controversy by not adjusting for height to define anemia and proposing as a cutoff point the 5th percentile of the hemoglobin distribution of all children in the DHS, assuming they were a healthy population ^16^.

Despite the need for local evidence on the behavior of hemoglobin at high altitude to reduce errors in the diagnosis of anemia in children who live in high altitude regions, no studies have been conducted in Peru on altitude adjustment that include biomarkers of iron and inflammation as recommended by the current literature ^15^; only one study in Puno determined the levels of biomarkers of iron and hepcidin in children under 3 years of age, and proposed not to use altitude correction ^17^. In this controversial scenario with different opinions, this study aimed to characterize iron status and propose to factors for adjusting hemoglobin for high altitude in children aged 6 to 8 months in Lima, Arequipa, Cusco and Puno.

**KEY MESSAGES**

Motivation for the study: There is controversy in Peru and the world about the application of adjustment factors to hemoglobin measurement at altitude.

Main findings: Hemoglobin increased exponentially according to altitude, while other iron biomarkers showed the opposite behavior evidencing the need for adjustment factors. Altitude adjustment values were estimated from 1000 to 4300 meters above sea level.

Implications: The adjustment values of hemoglobin estimated in the present study could be proposed to establish a more accurate diagnosis of anemia in children living in Lima, Arequipa, Cusco and Puno.

## MATERIALS AND METHODS

### Study design

Cross-sectional study with baseline data collected in a cohort of children 6 to 8 months of age attended in health facilities (EESS) of the Ministry of Health (MINSA). The original study, a population-based cohort, followed up the children in order to assess health care, iron intake and complementary feeding. The baseline assessment was conducted between November 2018 and November 2019.

### Population

The study population was children aged 6 to 8 months from 4 cities of different altitudes: Lima at 150 masl, Arequipa at 2335 masl, Cusco at 3400 masl and Puno at 3827 masl.

### Sample

We used all available data from the baseline assessment for our study, which consisted of 1744 infants and was collected non-probabilistically by convenience. The distribution of participants is shown in Figure S1 of the Supplementary Material.

The inclusion criteria were the following: a) healthy children 6 to 8 months of age attended in MINSA EESS in the referred cities (complete vaccination, complete growth and development controls (CRED), adequate complementary feeding and adequate iron intake); b) children of mothers who had normal pregnancy (without diseases); c) children of mothers who lived in the study area for at least two years; d) children born at term and single pregnancy; e) children without congenital diseases; and f) children whose parents agreed to participate in the study. The exclusion criteria were: a) children with an infectious disease one week before the evaluation.

### Procedures

The eligible children in the four cities were identified in the registry of the EESS where they attended. A health professional visited them to verify the inclusion criteria and invite them to participate in the study. After accepting to participate and signing the informed consent form, parents received an appointment for the evaluation, which consisted of an interview on socioeconomic and health information, the collection of a capillary blood sample from the child for hemoglobin measurement and the collection of a venous blood sample (3 mL) to determine iron and inflammatory biomarkers.

### Variables

Variables included age (6 months/7 months/8 months) and sex of children (male/female). We collected information about the mother regarding age (<18 years/18-29 years/>30 years), lives with partner (Yes/No), health insurance (Yes/No), received at least one prenatal checkup (Yes/No), prenatal iron supplement consumption (Yes/No), educational level (primary school or less, secondary school and higher education) and Ethnic self-identification (based on the 2017 Census question), categorized into: Mestizo, Quechua, Aymara, White, Amazonian native or indigenous, Afro-descendant, other). In addition, information was collected on household characteristics such as water connected to public network (Yes/No), drainage connected to public network (Yes/No) and gas stove (Yes/No).

In order to assess the altitude variable, an interviewer went to the child’s home and measured the altitude in meters at the main entrance of the home with a Garmin-Montana® GPS.

Hemoglobin (Hb) was measured by health personnel standardized in the technique. A capillary blood sample was taken with a calibrated HemoCue® Hb 201 portable hemoglobinometer.

Likewise, trained health personnel obtained the venous blood sample from the children. To do so, the mother was asked to sit the child on her lap, hug him or her and leave one of their arms free to examine the veins. Then, the most pronounced vein was located in the elbow flexure and the puncture was performed with a 20G x 1" yellow needle, and 3 ml of blood was collected in a 5 mL BD Vacutainer serum tube with separation gel. The vials with serum samples were stored at -70 °C in Puno, Cusco, Arequipa and -30 °C in Lima. Then, the thawed serum samples were sent on dry ice (-20 °C) to Germany for analysis of iron, vitamin A and inflammatory biomarkers at the VitMin Lab, Baden-Württemberg-Willstaett; these samples were analyzed by automated ELISA (s-ELISA) ^18^. The cut-off values were as follows: i) ID by serum ferritin (SF), when the value was <12 μg/L and <30 μg/L in the presence of inflammation; ii) ID by soluble transferrin receptor (RsTf) when the value was >8.3 mg/L; iii) vitamin A deficiency when retinol-binding protein (RBP) was <0.7 μmol/L. In order to assess inflammation, we considered C-reactive protein (CRP) with values >5 mg/L indicating an early phase of an infection and α1-acid glycoprotein (AGP) values >1 g/L as an indicator of a chronic infection ^15^. We also evaluated total body iron (TBI) using two biomarkers: serum ferritin and soluble transferrin receptor, by applying the following equation ^19^:


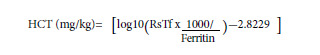


### Definition of the study groups

Four groups (models) were analyzed with the measurements of iron and inflammation biomarkers based on international recommendations ^15^ and the methodology applied in previous studies ^12^^-^^14^.

*Group 1*

Children with normal (>12µg/L) or adjusted (>30µg/L) SF in the presence of inflammation (CRP>5mg/L).

*Group 2*

Children without inflammation (CRP≤5mg/L), with normal SF (>12µg/L) and normal RBP (≥0.7µmol/L). The criteria recommended by the Biomarkers Reflecting Inflammation and Nutritional Determinants of Anemia (BRINDA) group ^15^^)^ were applied to this group; therefore, it was considered the reference group for the adjustment proposal of the National Institute of Health (INS), called INS 2023.

*Group 3*

Children without inflammation (CRP ≤5mg/L) with normal SF (>12µg/L), with normal RsTf (≤8.3mg/L) and normal RBP (≥0.7µmol/L).

*Group 4*

All children were included without considering iron status or inflammation as previous studies ^12^^-^^14^.

### Statistical analysis

We determined the absolute and relative distribution of the characteristics of the participants, as well as the mean and standard deviation (SD) of the biomarkers of iron and inflammation; additionally, the 5th and 95th percentile for Hb were also established. All measurements were estimated after evaluation of normality and homoscedasticity and biomarkers were compared according to group and city by ANOVA with Bonferroni post-hoc test or Dunn test for Kruskal-Wallis with Bonferroni post-hoc test.

In addition, the mean and SD of the original hemoglobin values (without adjustment for altitude) were estimated according to altitude of residence per 100 masl, starting from 100 to 200 masl and from 2300 to 4200 masl. Then, the distribution of hemoglobin according to altitude was identified, and the assumptions of the linear, quadratic and exponential function models were evaluated to select the fitting equation model. Also, the estimates and confidence intervals for hemoglobin were determined for each group and altitude of residence, and the hemoglobin values were compared according to the CDC/WHO parameters ^8^ and group 2 of the present study.

Complementarily, changes in the concentration of SF, HCT and RsTf were analyzed according to altitude using Spearman’s Rho correlation coefficient. The analyses were performed in Stata v17.0 (Stata Corporation, College Station, Texas, USA) considering a significance level of 0.05.

### Ethical aspects

The research protocol was approved by the INS Ethics Committee with code OI-018-17 and was registered in the PRISA platform with code EI00000002746.

## RESULTS

The study included all available data from the baseline measurement, which totaled 1744 children aged 6 to 8 months. Table 1 shows the main characteristics of each study group, highlighting group 4 with the highest number of observations, which also shows that there was a higher proportion of children aged 6 months (48.1%), residing in Lima (29.0%) and Arequipa (28.8%). Of the participants’ mothers, 59.4% were between 18 and 29 years old, 55.6% had secondary education, 95% had access to water connected to the public network, 92% had access to sewage connected to the public network and 51.7% identified themselves as being of Quechua descent. The same characteristics were assessed by city and are shown in Table S1 of the Supplementary Material.

**Table 1:** Characteristics of children aged 6 to 8 months included in the study according to group.

Characteristics	G1		G2		G3		G4	
	n	%	n	%	N	%	n	%
Age								
6 months	696	50.8	567	48.5	473	49.5	838	48.1
7 months	412	30.1	372	31.8	299	31.3	545	31.3
8 months	261	19.1	230	19.7	184	19.2	361	20.7
Sex								
Female	692	50.5	591	50.6	493	51.6	826	47.4
Male	677	49.5	578	49.4	463	48.4	918	52.6
City of residence								
Arequipa	387	28.3	337	28.8	275	28.8	502	28.8
Cusco	317	23.2	263	22.5	213	22.3	401	23.0
Lima	430	31.4	371	31.7	315	32.9	506	29.0
Puno	235	17.2	198	16.9	153	16.0	335	19.2
Age of the mother								
<18 years	28	2.0	24	2.1	19	2.0	35	2.0
18-29 years	794	58.0	674	57.7	549	57.4	1036	59.4
>30 years	547	40.0	471	40.3	388	40.6	673	38.6
Lives with a partner								
Yes	1153	84.2	982	84.0	799	83.6	1482	85.0
No	216	15.8	187	16.0	157	16.4	262	15.0
Has health insurance								
Yes	899	65.7	768	65.7	625	65.4	1137	65.2
No	470	34.3	401	34.3	331	34.6	607	34.8
Received at least one prenatal checkup								
Yes	1359	99.3	1162	99.4	951	99.5	1729	99.1
No	10	0.7	7	0.6	5	0.5	15	0.9
Prenatal iron supplement intake								
Yes	1303	95.2	1114	95.3	918	96.0	1651	94.7
No	66	4.8	55	4.7	38	4.0	93	5.3
Mother’s educational level								
Primary school	80	5.8	71	6.1	53	5.5	106	6.1
Secondary school	758	55.4	638	54.6	502	52.5	969	55.6
Higher education	531	38.8	460	39.3	401	41.9	669	38.4
Ethnic self-identification								
Mestizo	552	40.3	481	41.1	404	42.3	694	39.8
Quechua	697	50.9	586	50.1	475	49.7	901	51.7
Aimara	74	5.4	64	5.5	52	5.4	98	5.6
White	23	1.7	20	1.7	14	1.5	27	1.6
Amazonian native or indigenous,	14	1.0	11	0.9	8	0.8	15	0.9
Black/mulatto/zambo/Afro-Peruvian	4	0.3	3	0.3	2	0.2	4	0.2
Other	5	0.4	4	0.3	1	0.1	5	0.3
Water connected to public network								
Yes	1303	95.2	1113	95.2	912	95.4	1657	95.0
No	66	4.8	56	4.8	44	4.6	87	5.0
Drainage connected to public network								
Yes	1266	92.5	1083	92.6	890	93.1	1605	92.0
No	103	7.5	86	7.4	66	6.9	139	8.0
Gas stove								
Yes	1327	96.9	1135	97.1	931	97.4	1693	97.1
No	42	3.1	34	2.9	25	2.6	51	2.9
Total	1369	100.0	1169	100.0	956	100.0	1744	100.0

G1 (Group 1): children with normal (>12µg/L) or adjusted (>30µg/L) serum ferritin (SF) in the presence of inflammation (CRP>5mg/L). G2 (Group 2): Children without inflammation (CRP ≤5mg/L), with normal SF (>12µg/L) and normal retinol binding protein (RBP) (≥0.7µmol/L). G3 (Group 3): Children without inflammation (CRP ≤5mg/L) with normal SF (>12µg/L), with normal soluble transferrin receptor (RsTf) (≤8.3mg/L) and normal RBP (≥0.7µmol/L). G4 (Group 4): - All children were included regardless of iron status or presence of inflammation.

Regarding the iron status of the children, we found that, according to SF, the proportion of ID was 29.9% in Puno, 22.9% in Arequipa, 21% in Cusco and 15% in Lima; while applying RsTf, ID reached 38.8% of children aged 6 to 8 months in Puno, 29.7% in Cusco, 29.5% in Arequipa and 20.8% in Lima. In addition, we found that the proportion of children with elevated CRP was 18.7% in Cusco, 14.6% in Puno, 12.3% in Lima and 11.8% in Arequipa (Table 2).

**Table 2:** Iron deficiency, serum retinol deficiency and inflammation in children aged 6 to 8 months from Lima, Arequipa, Cusco and Puno.

Characteristics	Lima		Arequipa		Cusco		Puno	
	n	%	n	%	n	%	n	%
Iron deficiency								
According to serum ferritin adjusted by CRP ^a^	76	15.0	115	22.9	84	21.0	100	29.9
According to serum ferritin adjusted by AGP ^b^	69	13.6	115	22.9	81	20.2	92	27.5
According to soluble transferrin receptor	105	20.8	148	29.5	119	29.7	130	38.8
Serum retinol deficiency	70	13.8	72	14.3	72	18.0	63	18.8
Inflammation								
According to CRP ^a^	62	12.3	59	11.8	75	18.7	49	14.6
According to AGP ^b^	65	12.9	63	12.6	54	13.5	26	7.8

a C-reactive protein; ^b^ α-1-acid α-glycoprotein.

The average values of iron biomarkers are shown in Table 3 according to study group and city, and it can be seen that group 4 showed significantly lower values of SF and HCT, as well as, the highest value of RsTf (p<0.05); while group 3 showed the highest value of HCT and the lowest of RsTf (p<0.05). CRP values in groups 2 and 3 were equal and showed significant differences with groups 1 and 4 (p<0.05), while AGP only showed significant difference between group 3 and the rest of the groups (p<0.05). Furthermore, according to the cities, we found that the highest Hb values (without height correction) were reported in Puno and showed significant differences with Lima (p<0.05); however, the highest SF and HCT values were found in Lima with significant differences with the other three cities (p<0.05). The highest average CRP was found in Cusco and was significantly different from the other three cities (p<0.05).

**Table 3:** Measurements of biomarkers evaluated in children aged 6 to 8 months included in the study, according to group and city.

Characteristics	n	Hemoglobin (g/dL)		Serum ferritin (μg/L)	Soluble transferrin receptor (mg/L)	Total body iron (mg/kg)	Serum retinol (μmol/L)	CPR (mg/L)	AGP (g/L)
		Mean ± SD	5% - 95% Percentile	Mean ± SD	Mean ± SD	Mean ± SD	Mean ± SD	Mean ± SD	Mean ± SD
Group									
G1	1369	12.2 ±1.4	9.8 - 14.5	52.0±37.1^A^	7.1 ±1.8^A^	4.4 ± 3.4^A^	1.0 ±0.3^A^	3.8 ±10.4^A^	0.6 ±0.4^A^
G2	1169	12.2 ±1.4	9.8 - 14.5	51.0 ±36.9^B^	7.1 ±1.8^B,C^	4.3 ± 3.4^B,C^	1.0 ±0.3^A,B^	2.3 ±7.3^A^	0.6 ±0.3
G3	956	12.2 ±1.4	9.9 - 14.5	52.4 ±36.7^C^	6.5 ±1.1^A,B,D^	4.7 ± 3.3^A,B,D^	1.0 ±0.3^A,C^	2.3 ±7.3^A,B^	0.6 ±0.3^A,B^
G4	1744	12.1 ±1.5	9.7 - 14.4	42.7 ±37.4^A,B,C^	7.8 ±2.6^A,C,D^	3.2 ± 4.2^A,C,D^	1.0 ±0.3^B,C^	3.8 ±10.2^B^	0.6 ±0.4^B^
City									
Lima	506	10.7 ±1.1^a^	9.0 - 12.4	47.9 ±38.1^A^	7.1 ±2.3^A^	4.3 ±3.8^A^	1.00 ±0.3^A^	3.2 ±8.8^A^	0.6 ±0.4^A^
Arequipa	502	12.2 ±1.0^a^	10.6 - 13.8	44.9 ±40.6^B^	8.1 ±2.8^A^	2.6 ±4.4^A,B^	0.9 ±0.2^A^	3.1 ±9.2^B^	0.6 ±0.4^B^
Cusco	401	12.7 ±1.1^a^	10.9 - 14.5	44.1 ±37.4^A,C^	7.8 ±2.2^A^	3.5 ±3.9^A,B,C^	0.9 ±0.3	5.0 ±11.7^A,B,C^	0.6 ±0.4^C^
Puno	335	13.3 ±1.2^a^	11.2 - 15.3	30.0 ±27.6^A,B,C^	8.0 ±3.1^A^	2.0 ±4.1^A,C^	1.0 ±0.3	3.9 ±10.4^C^	0.5 ±0.3^A,B,C^

SD: standard deviation; CRP: C-reactive protein; AGP: α-1-acid glycoprotein; g/dL: grams per deciliter; μg/L: micrograms per liter; μmol/L: micromoles per liter; g/L: grams per liter.

a There was a significant difference between all regions according to the Bonferroni post-hoc comparison for Analysis of Variance (ANOVA) test. Capital letters in equal superscript represent statistically significant difference in vertical direction between groups of the biomarker evaluated, determined by Dunn's post-hoc test with Bonferroni adjustment for Kruskall-Wallis test.

G1 (Group 1): Children with normal (>12µg/L) or adjusted (>30µg/L) serum ferritin (SF) in the presence of inflammation (CRP>5mg/L); G2 (Group 2): Children without inflammation (CRP ≤5mg/L), with normal SF (>12µg/L) and normal retinol binding protein (RBP) (≥0.7µmol/L); G3 (Group 3): Children without inflammation (CRP ≤5mg/L) with normal SF (>12µg/L), normal soluble transferrin receptor (RsTf) (≤8.3mg/L) and normal RBP (≥0.7µmol/L); and G4 (Group 4): All children were included regardless of iron status or presence of inflammation.

Likewise, the mean and SD of Hb were determined for the individual data per 100 masl for the altitudes included in the study (Table S2 of the Supplementary Material) and the distribution (mean and confidence intervals) and trend of Hb per altitude were analyzed for the four study groups; we found a similar behavior in the four groups with an increase in Hb at higher altitudes (Figure S2 of the Supplementary Material).

After evaluating the Hb distribution functions, we identified that the exponential offered a better performance according to its coefficient of determination; the characteristics and values of the equations are shown in Tables S3 and S4 of the Supplementary Material. The equation of group 2 (INS 2023) was taken as reference: 10.34249 x (1.00007 ^ Alt).

Since a sub-analysis of the data available from a study whose objective was different from the present one was performed, we carried out an evaluation of the precision of the hemoglobin estimates for each city and predetermined group in order to guarantee the quality of the information (Table S5 of the Supplementary Material), which served as input for the identification of the exponential estimates according to altitudinal floor.

The mean Hb values of the four groups were estimated using exponential regression, and the absolute and relative difference of the estimates with the CDC/WHO adjustment and group 2 were calculated. In this comparison, negative differences of -0.8 to -1.7% were found between 1400 and 2600 masl; however, above 3000 to 4300 masl, positive differences ranging from 1.5 to 6.7% were found with an upward trend by altitude (Table 4). Table S6 of the Supplementary Material presents the behavior of the exponential increase in Hb estimates for each increase of 100 m asl for each of the groups evaluated.

**Table 4:** Exponential estimates of the mean hemoglobin values of children aged 6 to 8 months included in the study according to group by altitude of residence.

Altitude (masl )	Hemoglobin (g/dL)					Difference between G2 and CDC/WHO	
	G1 E (95% CI)	G2 E (95% CI)	G3 E (95% CI)	G4 E (95% CI)	CDC/WHO	Absolute (g/dL)	Relative (%)
100	10.6 (10.3-10.9)	10.4 (9.9-11.0)	10.5 (9.9-11.0)	10.4 (10.1-10.7)	11.0	0.6	5.5
1000	11.2 (11.0-11.4)	11.1 (10.7-11.5)	11.1 (10.7-11.6)	11.0 (10.8-11.2)	11.1	0.0	0.0
1100	11.3 (11.1-11.5)	11.2 (10.8-11.6)	11.2 (10.8-11.6)	11.1 (10.9-11.3)	11.2	0.0	0.0
1200	11.4 (11.2-11.6)	11.2 (10.9-11.6)	11.3 (10.9-11.7)	11.2 (11.0-11.4)	11.2	0.0	0.0
1300	11.5 (11.3-11.6)	11.3 (10.9-11.7)	11.4 (11.0-11.8)	11.2 (11.0-11.4)	11.3	0.0	0.0
1400	11.5 (11.3-11.7)	11.4 (11.0-11.8)	11.4 (11.1-11.8)	11.3 (11.1-11.5)	11.3	-0.1	-0.9
1500	11.6 (11.4-11.8)	11.5 (11.1-11.8)	11.5 (11.2-11.9)	11.4 (11.2-11.6)	11.4	-0.1	-0.9
1600	11.7 (11.5-11.8)	11.6 (11.2-11.9)	11.6 (11.3-11.9)	11.5 (11.3-11.7)	11.4	-0.2	-1.8
1700	11.8 (11.6-11.9)	11.6 (11.3-12.0)	11.7 (11.4-12.0)	11.6 (11.4-11.7)	11.5	-0.1	-0.9
1800	11.8 (11.7-12.0)	11.7 (11.4-12.0)	11.8 (11.4-12.1)	11.6 (11.5-11.8)	11.6	-0.1	-0.9
1900	11.9 (11.8-12.0)	11.8 (11.5-12.1)	11.8 (11.5-12.1)	11.7 (11.6-11.9)	11.7	-0.1	-0.9
2000	12.0 (11.8-12.1)	11.9 (11.6-12.2)	11.9 (11.6-12.2)	11.8 (11.6-11.9)	11.7	-0.2	-1.7
2100	12.1 (11.9-12.2)	12.0 (11.7-12.2)	12.0 (11.7-12.3)	11.9 (11.7-12.0)	11.8	-0.2	-1.7
2200	12.1 (12.0-12.3)	12.1 (11.8-12.3)	12.1 (11.8-12.3)	11.9 (11.8-12.1)	11.9	-0.2	-1.7
2300	12.2 (12.1-12.3)	12.1 (11.9-12.4)	12.2 (11.9-12.4)	12.0 (11.9-12.2)	12.0	-0.1	-0.8
2400	12.3 (12.2-12.4)	12.2 (12.0-12.4)	12.3 (12.0-12.5)	12.1 (12.0-12.2)	12.1	-0.1	-0.8
2500	12.4 (12.3-12.5)	12.3 (12.1-12.5)	12.3 (12.1-12.6)	12.2 (12.1-12.3)	12.2	-0.1	-0.8
2600	12.4 (12.4-12.5)	12.4 (12.2-12.6)	12.4 (12.2-12.6)	12.3 (12.2-12.4)	12.3	-0.1	-0.8
2700	12.5 (12.4-12.6)	12.5 (12.3-12.7)	12.5 (12.3-12.7)	12.4 (12.3-12.5)	12.5	0.0	0.0
2800	12.6 (12.5-12.7)	12.6 (12.4-12.7)	12.6 (12.4-12.8)	12.4 (12.3-12.5)	12.6	0.0	0.0
2900	12.7 (12.6-12.8)	12.7 (12.5-12.8)	12.7 (12.5-12.9)	12.5 (12.4-12.6)	12.7	0.0	0.0
3000	12.8 (12.7-12.9)	12.8 (12.6-12.9)	12.8 (12.6-13.0)	12.6 (12.5-12.7)	12.8	0.0	0.0
3100	12.9 (12.8-12.9)	12.8 (12.7-13.0)	12.9 (12.7-13.0)	12.7 (12.6-12.8)	13.0	0.2	1.5
3200	12.9 (12.9-13.0)	12.9 (12.8-13.1)	13.0 (12.8-13.1)	12.8 (12.7-12.9)	13.1	0.2	1.5
3300	13.0 (12.9-13.1)	13.0 (12.8-13.2)	13.0 (12.9-13.2)	12.9 (12.8-13.0)	13.3	0.3	2.3
3400	13.1 (13.0-13.2)	13.1 (12.9-13.3)	13.1 (12.9-13.3)	13.0 (12.9-13.0)	13.4	0.3	2.2
3500	13.2 (13.1-13.3)	13.2 (13.0-13.4)	13.2 (13.0-13.4)	13.0 (12.9-13.1)	13.6	0.4	2.9
3600	13.3 (13.2-13.4)	13.3 (13.1-13.5)	13.3 (13.1-13.5)	13.1 (13.0-13.2)	13.7	0.4	2.9
3700	13.4 (13.2-13.5)	13.4 (13.2-13.6)	13.4 (13.2-13.6)	13.2 (13.1-13.3)	13.9	0.5	3.6
3800	13.4 (13.3-13.6)	13.5 (13.2-13.7)	13.5 (13.2-13.8)	13.3 (13.2-13.4)	14.1	0.6	4.3
3900	13.5 (13.4-13.7)	13.6 (13.3-13.8)	13.6 (13.3-13.9)	13.4 (13.3-13.5)	14.2	0.6	4.2
4000	13.6 (13.5-13.8)	13.7 (13.4-14.0)	13.7 (13.4-14.0)	13.5 (13.3-13.6)	14.4	0.7	4.9
4100	13.7 (13.6-13.9)	13.8 (13.5-14.1)	13.8 (13.5-14.1)	13.6 (13.4-13.7)	14.6	0.8	5.5
4200	13.8 (13.6-14.0)	13.9 (13.5-14.2)	13.9 (13.5-14.2)	13.7 (13.5-13.8)	14.8	0.9	6.1
4300	13.9 (13.7-14.1)	14.0 (13.6-14.3)	14.0 (13.6-14.3)	13.8 (13.6-13.9)	15.0	1.0	6.7

CDC: Centers for Disease Control and Prevention; WHO: World Health Organization; masl.: meters above sea level; g/dL: grams per deciliter; E: exponential estimator; 95% CI: 95% confidence interval.

G1 (Group 1): Children with normal (>12µg/L) or adjusted (>30µg/L) serum ferritin (SF) in the presence of inflammation (CRP>5mg/L). G2 (Group 2): Children without inflammation (CRP ≤5mg/L), with normal SF (>12µg/L) and normal retinol binding protein (RBP) (≥0.7µmol/L). G3 (Group 3): Children without inflammation (CRP ≤5mg/L) with normal SF (>12µg/L), with normal soluble transferrin receptor (RsTf) (≤8.3mg/L) and normal RBP (≥0.7µmol/L). G4 (Group 4): All children were included regardless of iron status or presence of inflammation.

**Figure 1:**
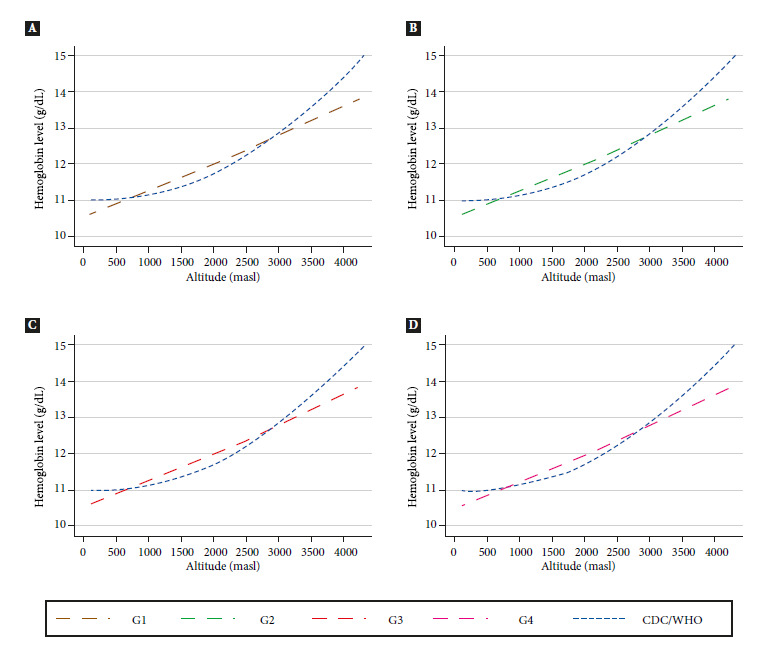
Behavior of the exponential estimates of the mean hemoglobin values of children aged 6 to 8 months included in the study according to group by altitude of residence: (a) Comparison of CDC/WHO estimates with Group 1. (b) Comparison of CDC/WHO estimates with Group 2. (c) Comparison of CDC/WHO estimates with Group 3. (d) Comparison of CDC/WHO estimates with Group 4. CDC: Centers for Disease Control and Prevention, WHO: World Health Organization, masl: meters above sea level.

Figure 1 shows the distribution of the exponential estimates of hemoglobin by altitude for the four study groups compared to the CDC reference curve. Overall, we found that for all groups, the Hb curves were above the CDC curve from less than 1000 masl up to approximately 3000 masl, then above this value the curves for all four groups were below the CDC curve which shows a greater inflection at higher altitude. When comparing the four curves and the CDC curve in a single graph (Figure S3 of the Supplementary Material), we found that the curves of groups 2 and 3 overlap; the curve of group 1 starts above group 2 and then at approximately 3500 masl falls below group 2. On the other hand, the curve of group 4 always remains below the curve of group 2.

**Figure 2A:**
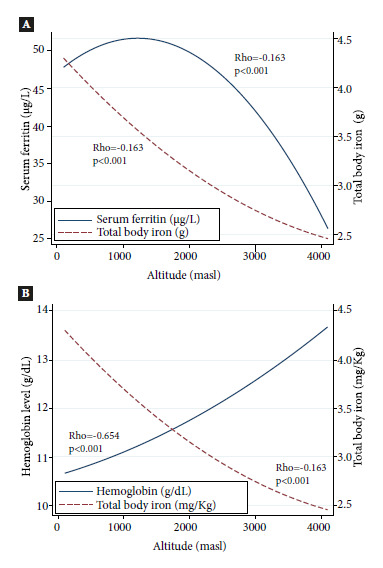
Correlation between serum ferritin and total body iron distribution by altitude in Peruvian children aged 6 to 8 months without inflammation [Group 2 - Children without inflammation, CRP > 5 mg/L with serum ferritin >12 μg/L and retinol-binding protein (RBP) ≥ 0.7 μmol/L]. **Figure 2B.** Correlation between hemoglobin and total body iron distribution by altitude in Peruvian children aged 6 to 8 months without inflammation [Group 2 - Children without inflammation, CRP > 5 mg/L with serum ferritin >12 μg/L and retinol-binding protein (RBP) ≥ 0.7 μmol/L]. masl: meters above sea level.

Figure 2A shows the effect of altitude on SF and HCT, in both cases there is a significant inverse relationship to the one with Hb; thus the higher the altitude, the lower the SF and HCT concentrations (p<0.001) in both cases. While Figure 2B shows an inverse relationship between Hb and HCT, there is a significant positive correlation between Hb and altitude (p<0.001); while HCT shows a negative correlation, also significant (p<0.001). Likewise, we found consistent behaviors of inverse relationship between RsTf with HCT and Hb with FS (Figure S4 and S5 of the Supplementary Material).

## DISCUSSION

This study aimed to characterize iron status and to propose hemoglobin adjustment factors for altitude. Regarding the first point, higher proportions of ID were found in children from cities with higher altitude (Cusco and Puno), in addition, they had lower mean values of SF, HCT and higher values of RsTf evidencing lower iron reserves at altitude. When analyzing hemoglobin by altitude, the results show an exponential increase in values in all children above 1000 masl, which could be explained as a physiological response to hypoxia and would evidence the need to adjust hemoglobin estimation for altitude.

It was necessary to define four study groups to contrast the results of the estimations in various situations in order to estimate the altitude adjustment factors. Thus, the first group considered the criteria used in EESS for the diagnosis of ID by SF in children with or without inflammation, the second group (INS 2023) was considered a reference group because it followed the recommendations of the BRINDA study ^15^. The third group in addition to the BRINDA criteria excluded children with ID by RsTf and the fourth group included all children without excluding children with ID or inflammation to reproduce the situation posed by authors proposing cut-off points using SIEN or ENDES data ^(12-14, 16)^. Other multinational studies seeking to verify hemoglobin adjustment factors for altitude also considered relatively similar criteria to define the study groups ^(10, 11)^.

The exponential model was selected for the hemoglobin adjustment equation for altitude, because it showed better statistical parameters. This approach agrees with that reported by Hurtado ^7^^)^ and Cohen ^20^^)^ in adult population, and Dirren ^9^^)^ and Bartolo ^14^^)^ in children; although it differs from others such as CDC ^8^, Ocas-Córdova ^13^, Sharma ^10^^)^ and Kanu ^11^ who proposed quadratic equations, and Miao ^21^^)^ who proposed a linear equation.

We found differences when comparing hemoglobin reference values between CDC/WHO and our study. Thus, the CDC/WHO values were lower between 1400 and 2600 masl, which could indicate an underestimation of anemia in this altitude range, while the CDC/WHO values above 3000 masl were higher, which would imply an overestimation of anemia from 3100 masl onwards. This trend of underestimation at altitudes between 1400 and 2600 masl and overestimation above 3000 masl agrees with that reported by Sharma *et al*. in preschool children and women ^10^ and Kanu *et al*. in school children ^11^.

Attributing characteristics of other populations such as those of Tibet to the inhabitants of high-altitude cities in southern Peru is another controversial aspect regarding altitude adjustment factors, because it would not be necessary ^22^. In this regard, differences have been reported regarding the increase of hemoglobin in populations living at high altitude with different ethnic origin, thus the population living in the Andean region has higher hemoglobin values than the population of Asia (Tibet) and Africa (Ethiopia) ^23^; therefore, the adaptation mechanisms of different populations would be based on gene expression, physiological adaptations and nutritional status ^24^. In addition, a reduction in plasma volume and higher levels of erythropoietin have been reported in the Andean population ^(^^25^^,^^26^.

The distribution of SF and HCT according to altitude showed a significant negative correlation (p<0.001), evidencing that altitude does not influence their values. Moreover, the curves of both biomarkers showed an opposite trend to the behavior of hemoglobin and RsTf which increased at higher altitude. The findings of lower SF, lower HCT, and higher RsTf values in children from three high-altitude cities suggest that they would not have sufficient reserves to increase hemoglobin ^27^. This finding could be explained by the fact that children aged 6 to 8 months require more iron to double blood volume ^3^^)^ and by the increased iron requirements due to hemoglobin adaptation to altitude ^28^. Although there are mechanisms that try to compensate iron metabolism, such as the regulation of hepcidin ^29^; it is also important to remember that when diets are poor in iron, as in Peru ^30^, these mechanisms are insufficient to cover iron needs, therefore children living in high-altitude areas would have an inadequate iron uptake and lower reserves to cover the higher demands ^31^.

Our results have implications for public health. First, the observed behavior of hemoglobin at high altitude would support the need to adjust hemoglobin estimates in high-altitude populations. Then, the hemoglobin adjustment values estimated in the present study could add evidence to help diagnose anemia more accurately at high altitude and contribute to more effective management of iron interventions in children in higher altitude cities.

One of the strengths of our study lies in the selection of healthy children who complied with their health and nutritional care; in addition, several biomarkers of iron and inflammation were included, which allowed the selection of children with normal iron status. Another strength was that data from four cities of different altitude were available to compare differences in hemoglobin status by altitude. Also, the biomarker analyses were performed in an international reference laboratory. The present study also has limitations. In that sense, our results only show baseline iron status in a cohort of children; however, it should be considered that the inclusion criteria allowed selection of children with good iron status. Besides, the biomarker estimates were performed in children 6 to 8 months of age, so those values cannot be directly extrapolated to other age groups. Our study has other limitations inherent to the sample design, for example we did not calculate a sample size to estimate hemoglobin values per 100 meters of altitude, but per selected city; this could affect the hemoglobin estimates. In this regard, we calculated the precision of the estimates and found acceptable values. In addition, the study sample was collected in a non-probabilistic manner, which could affect the external validity of the findings; however, it should be considered that the sample comes from a population-based cohort of children attended in EESS of MINSA and that the studies to elaborate reference standards emphasize more sampling strategies to guarantee healthy populations than representative populations ^32^. We also included four cities located at different altitudes, so the geographical representativeness could be limited; however, several studies with data from all over the country ^12^^-^^14^ show that altitude is the most influential variable for the behavior of hemoglobin. An additional limitation was that it was not possible to determine hepcidin levels in the participants; nevertheless, for the objectives of the present study, the BRINDA group ^15^ recommends determining only the biomarkers included in the study.

In conclusion, our study found higher proportions of ID in children from high altitude cities such as Cusco and Puno, and that their iron reserve values are lower compared to Lima. In addition, Hb showed an exponential distribution according to altitude, evidencing the need to apply an adjustment factor and based on the exponential model an adjustment equation was proposed for Hb in children residing in high altitude cities. The data suggest an underestimation of anemia between 1400-2600 masl and an overestimation between 3000-4300 masl when comparing the values estimated by CDC/WHO and our study. Larger studies on different age and physiological groups living at high altitude and in various regions are needed to corroborate these findings and should include information on dietary intake and iron metabolic balance.
