# Supplementary material for: Iron status and hemoglobin adjustment by altitude to define anemia in children aged 6 to 8 months living in Lima, Arequipa, Cusco and Puno
Source: Rev Peru Med Exp Salud Publica. 2023 Dec 18;40(4):395–405. doi: 10.17843/rpmesp.2023.404.12573 (PMC11138835; doi:10.17843/rpmesp.2023.404.12573)
Supplement: Supplementary material. — Available in the electronic version of the RPMESP. [file rpmesp-40-04-12573-s001.pdf]

MATERIAL SUPLEMENTARIO:

Estado de hierro y propuesta de ajuste de hemoglobina por altitud en niños de 6 a 8 meses residentes en Lima, Arequipa, Cusco y Puno.

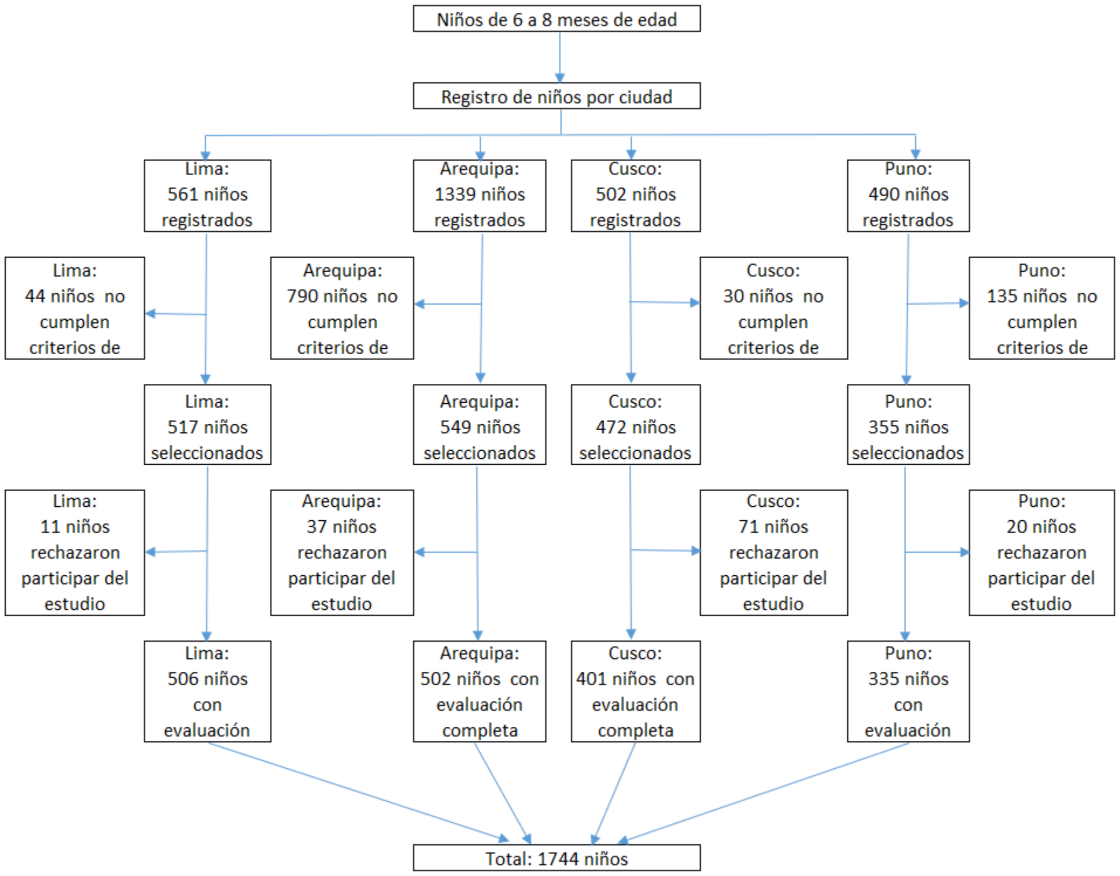

Figura S1. Flujograma de los niños de 6 a 8 meses que participaron del estudio.

Tabla S1. Distribución de las características de la muestra de estudio según ciudad de procedencia

	Arequipa n (%)	Cusco n (%)	Lima n (%)	Puno n (%)	Total n (%)
Edad					
6 meses	195 (38,8)	195 (48,6)	235 (46,4)	213 (63,6)	838 (48,1)
7 meses	160 (31,9)	130 (32,4)	166 (32,8)	89 (26,6)	545 (31,3)
8 meses	147 (29,3)	76 (19,0)	105 (20,8)	33 (9,9)	361 (20,7)
Sexo					
Femenino	228 (45,4)	197 (49,1)	239 (47,2)	162 (48,4)	826 (47,4)
Masculino	274 (54,6)	204 (50,9)	267 (52,8)	173 (51,6)	918 (52,6)
Edad de la madre					
<18 años	4 (0,8)	8 (2,0)	20 (4,0)	3 (0,9)	35 (2,0)
18 a 29 años	297 (59,2)	242 (60,3)	290 (57,3)	207 (61,8)	1036 (59,4)
≥30 años	201 (40,0)	151 (37,7)	196 (38,7)	125 (37,3)	673 (38,6)
Vive con pareja					
Sí	421 (83,9)	360 (89,8)	395 (78,1)	306 (91,3)	1482 (85,0)
No	81 (16,1)	41 (10,2)	111 (21,9)	29 (8,7)	262 (15)
Tenencia de seguro de salud					
Sí	289 (57,6)	291 (72,6)	345 (68,2)	212 (63,3)	1137 (65,2)
No	213 (42,4)	110 (27,4)	161 (31,8)	123 (36,7)	607 (34,8)
Recibió al menos un control prenatal					
Sí	499 (99,4)	399 (99,5)	502 (99,2)	329 (98,2)	1729 (99,1)
No	3 (0,6)	2 (0,5)	4 (0,8)	6 (1,8)	15 (0,9)
Consumo de suplemento de hierro prenatal					
Sí	492 (98,0)	386 (96,3)	481 (95,1)	292 (87,2)	1651 (94,7)
No	10 (2,0)	15 (3,7)	25 (4,9)	43 (12,8)	93 (5,3)
Educación de la madre					
Primaria	27 (5,4)	33 (8,2)	30 (5,9)	16 (4,8)	106 (6,1)
Secundaria	255 (50,8)	226 (56,4)	332 (65,6)	156 (46,6)	969 (55,6)
Superior	220 (43,8)	142 (35,4)	144 (28,5)	163 (48,7)	669 (38,4)
Raza					
Mestizo	215 (42,8)	80 (20,0)	382 (75,5)	17 (5,1)	694 (39,8)
Quechua	268 (53,4)	311 (77,5)	84 (16,6)	238 (71,0)	901 (51,7)
Aimara	14 (2,8)	1 (0,3)	5 (1,0)	78 (23,3)	98 (5,6)
Blanco	3 (0,6)	4 (1,0)	18 (3,6)	2 (0,6)	27 (1,6)
Nativo o indígena de la Amazonia	1 (0,2)	1 (0,3)	13 (2,6)	0 (0,0)	15 (0,9)
Negro/Mulato/Zambo/Afroperuano	0 (0,0)	0 (0,0)	4 (0,8)	0 (0,0)	4 (0,2)
Otros	1 (0,2)	4 (1,0)	0 (0,0)	0 (0,0)	5 (0,3)
Agua conectada a red pública					
Sí	490 (97,6)	376 (93,8)	503 (99,4)	288 (86,0)	1657 (95,0)
No	12 (2,4)	25 (6,2)	3 (0,6)	47 (14,0)	87 (5,0)
Desagüe conectado a red pública					
Sí	438 (87,3)	373 (93,0)	499 (98,6)	295 (88,1)	1605 (92,0)
No	64 (12,7)	28 (7,0)	7 (1,4)	40 (11,9)	139 (8)
Cocina a gas					
Sí	493 (98,2)	388 (96,8)	500 (98,8)	312 (93,1)	1693 (97,1)
No	9 (1,8)	13 (3,2)	6 (1,2)	23 (6,9)	51 (2,9)
Total	502 (100)	401 (100)	506 (100)	335 (100)	1744 (100)

Tabla S2. Mediciones de hemoglobina de los niños de 6 a 8 meses incluidos en el estudio según grupo, por altitud de residencia.

Altitud (msnm)	G1		G2		G3		G4	
	**n**	**Hb (g/dL) Media ±DE**	**n**	**Hb (g/dL) Media ±DE**	**n**	**Hb (g/dL) Media ±DE**	**n**	**Hb (g/dL) Media ±DE**
Desde 100 a 200	430	10,8 ±1,0	371	10,8 ±1,0	315	10,9 ±0,9	506	10,7 ±1,0
Desde 2300 a 2400	35	11,9 ±0,8	25	12,0 ±0,9	21	12,0 ±0,9	47	11,8 ±1,0
Desde 2400 a 2500	94	12,4 ±1,0	87	12,4 ±1,0	71	12,5 ±0,9	116	12,2 ±1,0
Desde 2500 a 2600	153	12,2 ±0,9	132	12,2 ±0,9	105	12,2 ±0,8	189	12,2 ±0,9
Desde 2600 a 2700	85	12,5 ±1,0	77	12,6 ±1,1	63	12,7 ±0,9	116	12,4 ±1,0
Desde 2700 a 2800	11	12,2 ±1,3	9	12,1 ±1,3	8	11,9 ±1,2	21	12,0 ±1,1
Desde 2800 a 2900	7	12,9 ±1,1	6	13,1 ±1,0	6	13,1 ±1,0	10	12,3 ±1,6
Desde 2900 a 3000	2	12,8 ±1,7	1	11,6 ±0,0	1	11,6 ±0,0	3	12,2 ±1,6
Desde 3000 a 3100	8	12,9 ±0,7	6	12,8 ±0,4	6	12,8 ±0,4	10	12,6 ±1,1
Desde 3100 a 3200	86	12,8 ±1,1	69	12,8 ±1,0	52	12,8 ±1,1	102	12,7 ±1,1
Desde 3200 a 3300	62	12,6 ±1,1	52	12,6 ±1,1	42	12,8 ±1,0	83	12,5 ±1,1
Desde 3300 a 3400	93	12,9 ±1,2	76	12,9 ±1,1	65	12,9 ±1,2	119	12,9 ±1,2
Desde 3400 a 3500	56	13,1 ±1,1	50	13,1 ±1,1	42	13,2 ±1,0	71	13,0 ±1,1
Desde 3500 a 3600	23	13,1 ±0,9	19	13,1 ±0,9	11	13,0 ±1,1	39	13,2 ±1,1
Desde 3600 a 3700	126	13,3 ±1,1	107	13,4 ±1,1	78	13,5 ±1,2	185	13,2 ±1,3
Desde 3700 a 3800	41	13,4 ±1,1	38	13,4 ±1,1	28	13,4 ±1,2	56	13,2 ±1,1
Desde 3800 a 3900	38	13,5 ±1,2	31	13,6 ±1,3	29	13,7 ±1,1	45	13,3 ±1,2
Desde 3900 a 4000	13	13,8 ±1,4	10	14,2 ±1,3	10	14,2 ±1,3	18	13,8 ±1,5
Desde 4000 a 4100	6	13,7 ±1,0	3	14,0 ±1,2	3	14,0 ±1,2	7	13,6 ±1,0
Desde 4100 a 4200	0	NA	0	NA	0	NA	1	13,6 ±0,0

- msnm: Metros sobre el nivel del mar, n: Cantidad de niños, Hb (g/dL): Hemoglobina medida en gramos por decilitro, DE: Desviación estándar, NA: No aplica por ausencia de casos.

- G1 (Grupo 1): Niños con ferritina sérica (FS) normal (≥ 12µg/L) o ajustada (>30µg/L) en presencia de inflamación (PCR>5mg/L). G2 (Grupo 2): Niños sin inflamación (PCR ≤5mg/L), con FS normal (≥ 12µg/L) y proteína fijadora de retinol (RBP) normal (≥0,7µmol/L). G3 (Grupo 3): Niños sin inflamación (PCR ≤5mg/L) con FS normal (≥ 12µg/L), con receptor soluble de transferrina (RsTf) normal (≤8,3mg/L) y RBP normal (≥0,7µmol/L). G4 (Grupo 4): Todos los niños fueron incluidos sin considerar el estado de hierro o presencia de inflamación.

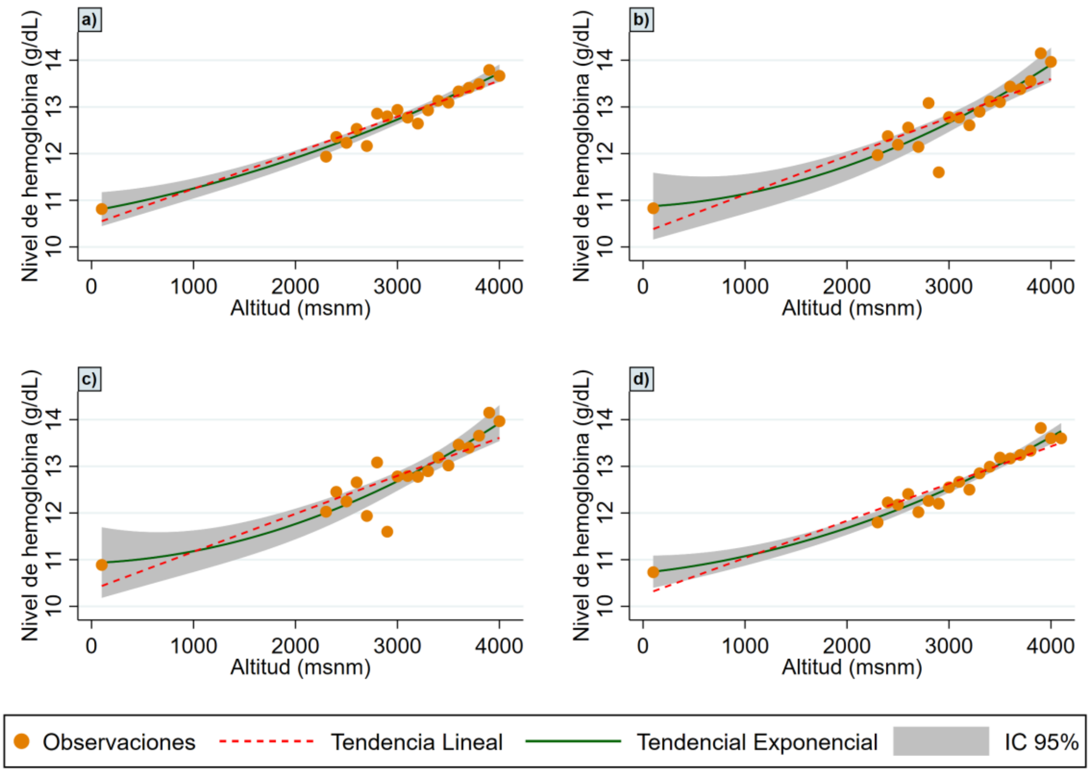

Figura S2. Distribución y tendencia de las mediciones de hemoglobina de los niños de 6 a 8 meses incluidos en el estudio según grupo por altitud geográfica de residencia. **a**: Grupo 1: Niños con ferritina sérica (FS) normal (≥ 12µg/L) o ajustada (>30µg/L) en presencia de inflamación (PCR>5mg/L), **b**: Grupo 2: Niños sin inflamación (PCR ≤5mg/L), con FS normal (≥ 12µg/L) y proteína fijadora de retinol (RBP) normal (≥0,7µmol/L), **c**: Grupo 3: Niños sin inflamación (PCR ≤5mg/L) con FS normal (≥ 12µg/L), con receptor soluble de transferrina (RsTf) normal (≤8,3mg/L) y RBP normal (≥0,7µmol/L), **d**: Grupo 4: Todos los niños fueron incluidos sin considerar el estado de hierro o presencia de inflamación

Tabla S3. Características de las funciones generadas por grupo para la estimación de valores de hemoglobina de los niños de 6 a 8 meses por altitud geográfica de residencia.

CARACTERÍSTICAS	G1			G2			G3			G4		
	GL	CM	r^2	GL	CM	r^2	GL	CM	r^2	GL	CM	r^2
Función lineal			0,923			0,782			0,762			0,907
Modelo	1	8,14		1	9,27		1	9,05		1	9,40	
Residuo	17	0,04		17	0,15		17	0,17		18	0,05	
Total	18	0,49		18	0,66		18	0,66		19	0,55	
Función Cuadrática			0,946			0,845			0,827			0,956
Modelo	2	4,17		2	5,01		2	4,91		2	4,95	
Residuo	16	0,03		16	0,11		16	0,13		17	0,03	
Total	18	0,49		18	0,66		18	0,66		19	0,55	
Función Exponencial			>0,999			0,999			0,999			>0,999
Modelo	2	1556,51		2	1552,59		2	1558,37		2	1609,26	
Residuo	17	0,03		17	0,14		17	0,15		18	0,04	
Total	19	163,87		19	163,55		19	164,18		20	160,96	

- GL: Grados de libertad, CM: Cuadrados medios, r^2: Coeficiente de determinación.
- G1 (Grupo 1): Niños con ferritina sérica (FS) normal (≥ 12µg/L) o ajustada (>30µg/L) en presencia de inflamación (PCR>5mg/L). G2 (Grupo 2): Niños sin inflamación (PCR ≤5mg/L), con FS normal (≥ 12µg/L) y proteína fijadora de retinol (RBP) normal (≥0,7µmol/L). G3 (Grupo 3): Niños sin inflamación (PCR ≤5mg/L) con FS normal (≥ 12µg/L), con receptor soluble de transferrina (RsTf) normal (≤8,3mg/L) y RBP normal (≥0,7µmol/L). G4 (Grupo 4): Todos los niños fueron incluidos sin considerar el estado de hierro o presencia de inflamación.

Tabla S4. Valores de las ecuaciones según función y grupo para la estimación de valores de hemoglobina de los niños de 6 a 8 meses por altitud geográfica de residencia.

FUNCIÓN	G1	G2	G3	G4
Lineal	10,47569 + (0,0007719 x Alt)	10,30194 + (0,0008237 x Alt)	10,35547 + (0,0008139 x Alt)	10,24272 + (0,0007961 x Alt)
Cuadrática	(0,0004 x Alt) + (0,0000000847 x Alt) + 10,76927	(0,0001127 x Alt) + (0,000000162 x Alt) + 10,86323	(0,000088 x Alt) + (0,000000165 x Alt) + 10,92851	(0,000233 x Alt) + (0,000000124 x Alt) + 10,71838
Exponencial	10,53688 x (1,000064 ^ Alt)	10,34249 x (1,00007 ^ Alt)	10,39253 x (1,000069 ^ Alt)	10,30093 x (1,000067 ^ Alt)

- Alt: Altitud en metros sobre el nivel del mar.
- G1 (Grupo 1): Niños con ferritina sérica (FS) normal (≥ 12µg/L) o ajustada (>30µg/L) en presencia de inflamación (PCR>5mg/L). G2 (Grupo 2): Niños sin inflamación (PCR ≤5mg/L), con FS normal (≥ 12µg/L) y proteína fijadora de retinol (RBP) normal (≥0,7µmol/L). G3 (Grupo 3): Niños sin inflamación (PCR ≤5mg/L) con FS normal (≥ 12µg/L), con receptor soluble de transferrina (RsTf) normal (≤8,3mg/L) y RBP normal (≥0,7µmol/L). G4 (Grupo 4): Todos los niños fueron incluidos sin considerar el estado de hierro o presencia de inflamación.

Tabla S5. Determinación de la precisión en la estimación de hemoglobina según grupo evaluado y ciudad de origen.

	**n**	**Desviación Estándar Identificada**	**Precisión**	**Rango de precisión de la Desviación Estándar**
Grupos				
G1	1369	1,4	0,074	1,326; 1,474
G2	1169	1,4	0,080	1,320; 1,480
G3	956	1,4	0,089	1,311; 1,489
G4	1744	1,5	0,070	1,430; 1,570
Ciudad				
Lima	506	1,1	0,096	1,004; 1,196
Arequipa	502	1,0	0,087	0,913; 1,087
Cusco	401	1,1	0,108	0,992; 1,208
Puno	335	1,2	0,129	1,071; 1,329

G1 (Grupo 1): Niños con ferritina sérica (FS) normal (≥ 12µg/L) o ajustada (>30µg/L) en presencia de inflamación (PCR>5mg/L). G2 (Grupo 2): Niños sin inflamación (PCR ≤5mg/L), con FS normal (≥ 12µg/L) y proteína fijadora de retinol (RBP) normal (≥0,7µmol/L). G3 (Grupo 3): Niños sin inflamación (PCR ≤5mg/L) con FS normal (≥ 12µg/L), con receptor soluble de transferrina (RsTf) normal (≤8,3mg/L) y RBP normal (≥0,7µmol/L). G4 (Grupo 4): Todos los niños fueron incluidos sin considerar el estado de hierro o presencia de inflamación.

Tabla S6. Incremento exponencial en la estimación de los valores medios de hemoglobina por altitud geográfica de residencia respecto al piso altitudinal previo (previos 100 metros sobre nivel del mar).

Altitud	Incremento			
	G1	G2	G3	G4
De 1000 a 1100	0.072	0.078	0.077	0.074
De 1100 a 1200	0.073	0.078	0.077	0.075
De 1200 a 1300	0.073	0.079	0.078	0.075
De 1300 a 1400	0.074	0.079	0.079	0.076
De 1400 a 1500	0.074	0.080	0.079	0.076
De 1500 a 1600	0.075	0.080	0.080	0.077
De 1600 a 1700	0.075	0.081	0.080	0.078
De 1700 a 1800	0.076	0.082	0.081	0.078
De 1800 a 1900	0.076	0.082	0.081	0.079
De 1900 a 2000	0.077	0.083	0.082	0.079
De 2000 a 2100	0.077	0.083	0.082	0.080
De 2100 a 2200	0.078	0.084	0.083	0.080
De 2200 a 2300	0.078	0.084	0.084	0.081
De 2300 a 2400	0.079	0.085	0.084	0.081
De 2400 a 2500	0.079	0.086	0.085	0.082
De 2500 a 2600	0.080	0.086	0.085	0.082
De 2600 a 2700	0.080	0.087	0.086	0.083
De 2700 a 2800	0.081	0.087	0.086	0.083
De 2800 a 2900	0.081	0.088	0.087	0.084
De 2900 a 3000	0.082	0.089	0.088	0.085
De 3000 a 3100	0.082	0.089	0.088	0.085
De 3100 a 3200	0.083	0.090	0.089	0.086
De 3200 a 3300	0.083	0.091	0.089	0.086
De 3300 a 3400	0.084	0.091	0.090	0.087
De 3400 a 3500	0.084	0.092	0.091	0.088
De 3500 a 3600	0.085	0.092	0.091	0.088
De 3600 a 3700	0.085	0.093	0.092	0.089
De 3700 a 3800	0.086	0.094	0.093	0.089
De 3800 a 3900	0.087	0.094	0.093	0.090
De 3900 a 4000	0.087	0.095	0.094	0.091
De 4000 a 4100	0.088	0.096	0.095	0.091
De 4100 a 4200	0.088	0.096	0.095	0.092
De 4200 a 4300	0.089	0.097	0.096	0.092

G1 (Grupo 1): Niños con ferritina sérica (FS) normal (≥ 12µg/L) o ajustada (>30µg/L) en presencia de inflamación (PCR>5mg/L). G2 (Grupo 2): Niños sin inflamación (PCR ≤5mg/L), con FS normal (≥ 12µg/L) y proteína fijadora de retinol (RBP) normal (≥0,7µmol/L). G3 (Grupo 3): Niños sin inflamación (PCR ≤5mg/L) con FS normal (≥ 12µg/L), con receptor soluble de transferrina (RsTf) normal (≤8,3mg/L) y RBP normal (≥0,7µmol/L). G4 (Grupo 4): Todos los niños fueron incluidos sin considerar el estado de hierro o presencia de inflamación.

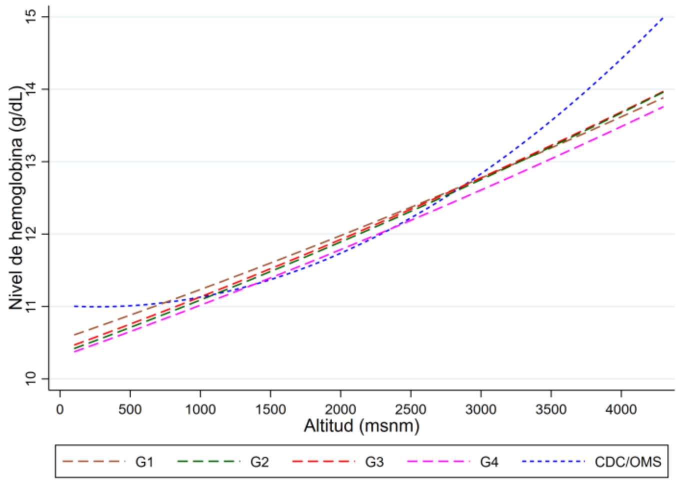

Figura S3. Comportamiento de las estimaciones exponenciales de los valores medios de hemoglobina de los niños de 6 a 8 meses incluidos en el estudio según grupo por altitud geográfica de residencia.

G1 (Grupo 1): Niños con ferritina sérica (FS) normal (≥ 12µg/L) o ajustada (>30µg/L) en presencia de inflamación (PCR>5mg/L). G2 (Grupo 2): Niños sin inflamación (PCR ≤5mg/L), con FS normal (≥ 12µg/L) y proteína fijadora de retinol (RBP) normal (≥0,7µmol/L). G3 (Grupo 3): Niños sin inflamación (PCR ≤5mg/L) con FS normal (≥ 12µg/L), con receptor soluble de transferrina (RsTf) normal (≤8,3mg/L) y RBP normal (≥0,7µmol/L). G4 (Grupo 4): Todos los niños fueron incluidos sin considerar el estado de hierro o presencia de inflamación.

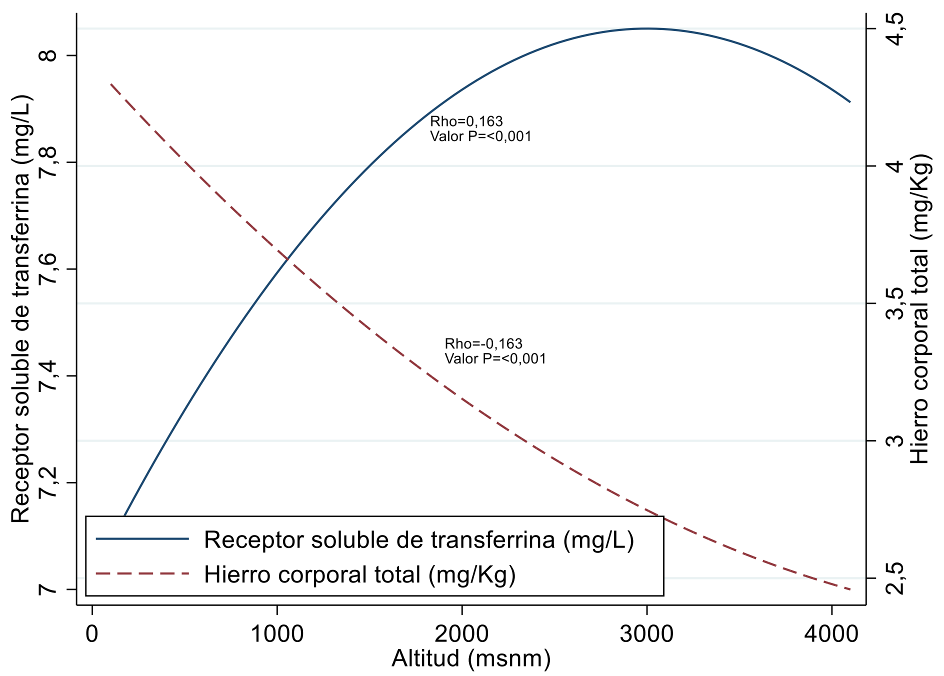

Figura S4. Correlación entre la distribución de receptor soluble de transferrina y hierro corporal total por altitud en niños peruanos de 6 a 8 meses sin inflamación. Niños del Grupo 2: Sin inflamación (PCR ≤5mg/L), con FS normal (≥ 12µg/L) y proteína fijadora de retinol (RBP) normal (≥0,7µmol/L).

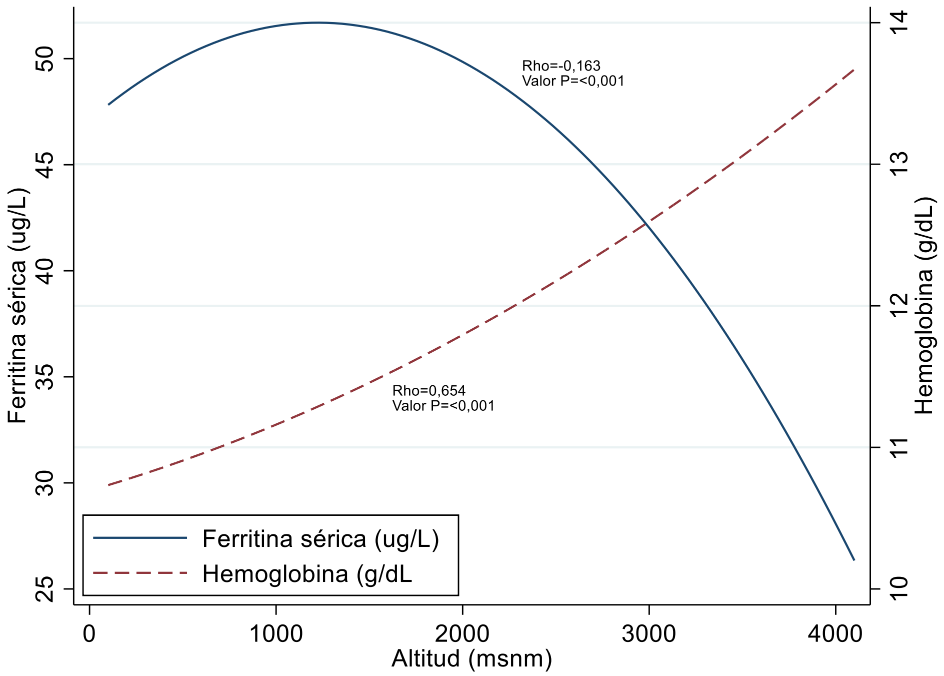

Figura S5. Correlación entre la distribución de ferritina sérica y hemoglobina por altitud en niños peruanos de 6 a 8 meses sin inflamación. Niños del Grupo 2: Sin inflamación (PCR ≤5mg/L), con FS normal (≥ 12µg/L) y proteína fijadora de retinol (RBP) normal (≥0,7µmol/L).
