# Supplementary material for: Iron status and hemoglobin adjustment by altitude to define anemia in children aged 6 to 8 months living in Lima, Arequipa, Cusco and Puno
Source: Rev Peru Med Exp Salud Publica. 2023 Dec 18;40(4):395–405. doi: 10.17843/rpmesp.2023.404.12573 (PMC11138835; doi:10.17843/rpmesp.2023.404.12573)
Supplement: Translation

# Translation: Estado de hierro y propuesta de ajuste de hemoglobina por altitud en niños de 6 a 8 meses residentes en Lima, Arequipa, Cusco y Puno

**Authors:** Juan Pablo Aparco, Gabriela Santos-Antonio, William Bautista-Olortegui, Katherine Alvis-Chirinos, Patricia Velarde-Delgado, Paul Hinojosa-Mamani, Gilmer Solis-Sanchez, Flor Eliana Santa Cruz, Nelly Zavaleta

**Affiliations:** 1 Centro Nacional de Alimentación, Nutrición y Vida Saludable, Instituto Nacional de Salud (INS), Lima, Perú.; 2 Dirección General de Intervenciones Estratégicas en Salud Pública, Ministerio de Salud (MINSA), Lima, Perú.; 3 Instituto de Investigación Nutricional (IIN), Lima, Perú.

**Keywords:** Niños, Anemia, Altitud, Deficiencia de Hierro, Perú

## Abstract

**Objetivos.:**

Caracterizar el estado del hierro y proponer factores de ajuste de hemoglobina por altitud, en niños de 6 a 8 meses de Lima, Arequipa, Cusco y Puno.

**Materiales y métodos.:**

Estudio transversal en niños de 6 a 8 meses de edad en cuatro ciudades, se midió hemoglobina y otros biomarcadores de hierro, Proteína C reactiva (PCR), entre otros. Para estimar la ecuación de ajuste, se aplicó una regresión exponencial y excluimos a los niños con deficiencia de hierro (DH) y/o inflamación.

**Resultados.:**

Las proporciones de DH fueron mayores en Puno y Arequipa, mientras que la inflamación no superó el 19% en ninguna de las ciudades. La hemoglobina mostró un incremento exponencial a mayor altitud. La ecuación de ajuste fue: 10,34249 x (1,00007 ^ Alt).

**Conclusiones.:**

Los niños residentes en Arequipa y Puno mostraron mayores proporciones de DH y menores reservas de hierro; además el aumento de la hemoglobina por altitud fue exponencial evidenciando la necesidad de ajustar la hemoglobina en altura.

## INTRODUCCIÓN

La anemia es un problema de salud pública mundial y afecta a casi dos mil millones de personas, siendo más vulnerables los niños de países en desarrollo ^(^^1^. La deficiencia de hierro (DH), las hemoglobinopatías y la malaria son consideradas las principales causas de la anemia ^2^. Las consecuencias de la anemia sobre la salud y el desarrollo se han descrito previamente ^3^^,^^4^ y los efectos continuarían a lo largo de la vida ^(^^5^. El diagnostico de anemia se realiza aplicando puntos de corte considerando la edad, sexo y estado fisiológico del individuo, así como factores de ajuste por hábito tabáquico y altitud ^6^.

En 1945, Hurtado planteó corregir las estimaciones de hemoglobina en altitud, porque este biomarcador aumenta como respuesta adaptativa a la baja presión de oxígeno en la altura y propuso un factor de ajuste basado en datos de varones adultos peruanos ^7^. En 1989, el Centro de Control de Enfermedades de Estados Unidos (CDC, por sus siglas en inglés) publicó factores de ajuste por altitud empleando datos de niños entre 2 a 4 años y residentes en altitudes hasta los 2150 metros sobre el nivel del mar (msnm) y para estimar factores de ajuste a mayor altitud usaron los datos de Hurtado ^(^^8^. Luego en 1994, Dirren empleando datos de niños ecuatorianos menores de 5 años encontró un factor de ajuste similar al de CDC ^9^. En el año 2001, la Organización Mundial de la Salud (OMS) adoptó los factores de ajuste por altitud ^6^ publicados por CDC; sin embargo, la evidencia más actual muestra una posible sobreestimación de anemia en residentes a mayores altitudes ^(10, 11)^, por lo que la OMS inició la revisión del ajuste por altitud y las directrices mundiales de anemia.

En el Perú, Gonzales-Rengifo planteó no aplicar ajuste por altitud a la hemoglobina en población de la región sur porque estarían mejor adaptados a la altitud tal como ocurre en el Tibet ^(^^12^; más adelante, este mismo autor elaboró un factor de ajuste con datos del Sistema de Información del Estado Nutricional (SIEN) ^(^^13^. Asimismo, Bartolo et al., propusieron un factor de ajuste usando la Encuesta Nacional de Demografía y Salud (ENDES) de 2015 ^14^; sin embargo, en ambos estudios los modelos solo se basaron en hemoglobina capilar sin evaluar el estado de hierro de los niños u otros factores que podrían elevar el nivel de hemoglobina como la inflamación ^15^. Recientemente, Accinelli *et al.*, con datos de la ENDES de2017, reavivó la controversia al no usar ajuste por altura para definir anemia y proponer como punto de corte el percentil5 de la distribución de hemoglobina de todos los niños de la ENDES, asumiendo que eran una población sana ^16^.

Pese a la necesidad de contar con evidencia local del comportamiento de la hemoglobina en altitud para reducir errores en el diagnóstico de anemia en niños de ciudades de altura; en el Perú, no se han realizado estudios sobre ajuste por altitud que incluyan biomarcadores de hierro e inflamación como lo recomienda la literatura actual ^15^; solo un estudio en Puno determinó los niveles de biomarcadores dehierro y hepcidina en menores de 3 años, y planteó no usar corrección por altura ^17^. En este escenario controversial con varias posturas, se planteó el presente estudio con el objetivo de caracterizar el estado de hierro y proponer factores de ajuste de hemoglobina por altitud en niños de 6 a 8 meses de Lima, Arequipa, Cusco y Puno.

**MENSAJE CLAVE**

Motivación para realizar el estudio: En el Perú y el mundo existe controversia sobre la aplicación de factores de ajuste a la medición de hemoglobina en altitud.

Principales hallazgos: La hemoglobina mostró un comportamiento exponencial de incremento según altitud, mientras que otros biomarcadores de hierro mostraron un comportamiento contrario evidenciando la necesidad de factores de ajuste. Se estimaron valores de ajuste por altitud desde los 1000 hasta los 4300 metros sobre el nivel del mar.

Implicancias: Los valores de ajuste de hemoglobina estimados en el presente estudio podrían contribuir como una propuestapara establecer un diagnóstico más preciso de anemia en niños residentes de Lima, Arequipa, Cusco y Puno.

## MATERIALES Y MÉTODOS

### Diseño del estudio

Estudio transversal con datos basales recolectados en una cohorte en niños de 6 a 8 meses de edad atendidos en establecimientos de salud (EESS) del Ministerio de Salud (MINSA). El estudio original, una cohorte de base poblacional, realizó un seguimiento de los niños para observar las atenciones de salud, consumo de hierro y alimentación complementaria. La evaluación basal se realizó entre noviembre de 2018 y noviembre de 2019.

### Población

La población de estudio fueron niños de 6 a 8 meses de 4 ciudades de diferentes altitudes: Lima a 150 msnm, Arequipa a 2335 msnm, Cusco a 3400 m s. n. m. y Puno a 3827 msnm.

### Muestra

Para este análisis se utilizó la totalidad de datos disponibles de la evaluación basal, constituida por 1744 infantes y fue recolectada de forma no probabilística por conveniencia. La distribución de los participantes se muestra en la figura S1 del Material Suplementario.

Los criterios de inclusión fueron: a) Niños sanos de 6 a 8 meses de edad atendidos en EESS del MINSA de las ciudades referidas (vacunación completa, controles de crecimiento y desarrollo (CRED) completos, alimentación complementaria adecuada y consumo de hierro adecuado); b) hijos de madres que tuvieron gestación normal (sin enfermedades); c) hijos de madres que vivieron en el área de estudio por lo menos dos años; d) niños nacidos a término y de gestación única; e) niños sin enfermedades congénitas; y f) niños cuyos padres aceptaron participar del estudio. El criterio de exclusión fue: a) niños con alguna enfermedad infecciosa una semana antes de la evaluación.

### Procedimientos

En las cuatro ciudades los niños elegibles fueron identificados en el registro del EE.SS. donde acudían, luego un profesional de salud los visitó para verificar los criterios de inclusión e invitarlos a participar en el estudio. Después de la aceptación y firma del consentimiento informado, los padres recibieron una cita para la evaluación que consistió en una entrevista sobre información socioeconómica y de salud, la toma de una muestra de sangre capilar del niño, para medición de hemoglobina y la toma de una muestra de sangre venosa (3 mL) para determinar los biomarcadores de hierro e inflamatorios.

### Variables

Las variables incluían la edad (6 meses/7 meses/8 meses) y sexo de los niños (masculino/femenino). Se recolectó información de la madre sobre la edad (<18 años/18-29 años/>30 años), vive con pareja (Sí/No), tenencia de seguro de salud (Sí/No), recibió al menos un control prenatal (Sí/No), consumo de suplemento de hierro prenatal (Si/No), nivel educativo (primaria o menos, secundaria y superior) y Autoidentificación étnica (en base a la pregunta del Censo 2017), se categorizó en: mestizo, quechua, aimara, blanco, nativo o indígena de la amazonia, afrodescendiente, otros). Además, se recolectó información sobre las características del hogar como agua conectada a red pública (Sí/No), desagüe conectado a red pública (Sí/No) y de cocina a gas (Sí/No).

Para la variable altitud un encuestador se trasladó a la vivienda del niño y en la entrada principal del hogar midió la altitud en metros con un GPS Garmin-Montana®.

Para determinar los indicadores bioquímicos, la hemoglobina (Hb) fue medida por personal de salud estandarizados en la técnica. Se tomó una muestra de sangre capilar con un hemoglobinómetro portátil calibrado HemoCue® Hb 201.

Asimismo, personal de salud entrenado tomó la muestra de sangre venosa en los niños, para ello se pidió a la madre que siente al niño o niña en su regazo, lo abrace y deje libre uno de sus brazos para examinarle las venas. A continuación, se procedió a ubicar la vena más pronunciada en la flexura del codo y con una aguja amarilla 20G x 1” se procedió a realizar la punción y se recolectó 3 ml de sangre en un tubo de suero BD Vacutainer de 5 mL con gel de separación. Los viales con muestras de suero se almacenaron a -70 °Cen Puno, Cusco, Arequipa y -30 °C en Lima. Luego, las muestras de suero concongeladas se enviaron en hielo seco (-20 °C) a Alemania para el análisis de hierro, vitamina A y biomarcadores inflamatorios en el laboratorio VitMin Lab, Baden-Württemberg-Willstaett; dichas muestras se analizaron a través de la técnica de ELISA automatizado (s-ELISA) ^18^. Los valores de corte fueron los siguientes: i) DH por ferritina sérica (FS), cuando el valor era <12 μg/L y <30 μg/L en presencia de inflamación; ii) DH por receptor soluble de transferrina (RsTf) cuando el valor era >8,3 mg/L; iii) deficiencia de vitamina A cuando la proteína fijadora de retinol (RBP por sus siglas en inglés) era <0,7 μmol/L. Para inflamación se consideraron la proteína C reactiva (PCR) con valores >5 mg/L que indican una fase temprana de una infección y en α1-glicoproteína ácida (AGP) valores >1 g/L como indicador de una infección crónica ^(^^15^. También evaluamos el hierro corporal total (HCT, TBI por sus siglas en inglés) utilizando dos biomarcadores: ferritina sérica y receptor soluble de transferrina, aplicando la siguiente ecuación ^19^:



### Definición de los grupos de estudio

Con las mediciones de los biomarcadores de hierro e inflamación se analizaron cuatro grupos (modelos) basados en las recomendaciones internacionales ^15^ y la metodología

aplicada en estudios previos ^12^^-^^14^.

*Grupo 1*

Niños con FS normal (>12µg/L) o ajustada (>30µg/L) en presencia de inflamación (PCR>5mg/L).

*Grupo 2*

Niños sin inflamación (PCR≤5mg/L), con FS normal (>12µg/L) y RBP normal (≥0,7µmol/L). En este grupo se aplicaron los criterios recomendados por el grupo *Biomarkers Reflecting Inflammation and Nutritional Determinants of Anemia* (BRINDA) ^15^ por lo que, se consideró el grupo de referencia para la propuesta de ajuste del Instituto Nacional de Salud (INS), denominado INS 2023.

*Grupo 3*

Niños sin inflamación (PCR ≤5mg/L) con FS normal (>12µg/L), con RsTf normal (≤8,3mg/L) y RBP normal (≥0,7µmol/L).

*Grupo 4*

Todos los niños fueron incluidos sin considerar el estado de hierro o presencia de inflamación como procedieron estudios previos ^12^^-^^14^.

### Análisis estadístico

Se determinó la distribución absoluta y relativa de las características de los participantes, además se identificó la media y desviación estándar (DE) de los biomarcadores de hierro e inflamación, adicionalmente, se estableció el percentil 5 y 95 para la Hb. Todas las mediciones se estimaron previa evaluación de normalidad y homocedasticidad y se compararon los biomarcadores según grupo y ciudad mediante ANOVA con prueba *post-hoc* de Bonferroni o prueba Dunn para Kruskal-Wallis con *post-hoc* de Bonferroni.

Además, se estimó la media y DE de los valores originales de hemoglobina (sin ajuste por altitud) según altitud de residencia por cada 100 msnm iniciando entre los 100 a 200 msnm y de los 2300 a 4200 msnm. Luego, se identificó la distribución de hemoglobina según altitud y para seleccionar el modelo de ecuación de ajuste se procedió a evaluar los supuestos de los modelos de función lineal, cuadrática y exponencial. También, se determinaron las estimaciones e intervalos de confianza para la hemoglobina por cada grupo y altitudes de residencia, y se compararon los valores de hemoglobina según los parámetros del CDC/OMS ^8^ y el grupo 2 del presente estudio.

Complementariamente, se analizaron los cambios en la concentración de FS, HCT y RsTf según altitud mediante el coeficiente de correlación Rho de Spearman. Los análisis se realizaron en Stata v17.0 (Stata Corporation, College Station, Texas, USA) considerando un nivel de significancia de 0,05.

### Aspectos éticos

El protocolo de investigación fue aprobado por el Comité de Ética del INS con el Código OI-018-17 y se registró en la plataforma PRISA con código EI00000002746.

## RESULTADOS

El estudio incluyó todos los datos disponibles de la medición basal, que totalizó 1744 niños de 6 a 8 meses de edad. La Tabla 1 muestra las principales características de cada grupo de estudio, destacando el grupo 4 con mayor número de observaciones, además se aprecia que hubo una mayor proporción de niños de 6 meses (48,1%), residentes en Lima (29,0%) y Arequipa (28,8%). El 59,4% de las madres de los participantes tenía entre 18 y 29 años, 55,6% tenía educación secundaria, 95% acceso a agua conectada a red pública, 92% desagüe conectado a red pública y el 51,7% se identificaba como descendiente de quechuas. Las mismas características se evaluaron por ciudad y se muestran en la Tabla S1 del Material Suplementario.

**Tabla 1:** Características de los niños de 6 a 8 meses incluidos en el estudio según grupo.

Características	G1		G2		G3		G4	
	n	%	n	%	N	%	n	%
Edad								
6 meses	696	50,8	567	48,5	473	49,5	838	48,1
7 meses	412	30,1	372	31,8	299	31,3	545	31,3
8 meses	261	19,1	230	19,7	184	19,2	361	20,7
Sexo								
Femenino	692	50,5	591	50,6	493	51,6	826	47,4
Masculino	677	49,5	578	49,4	463	48,4	918	52,6
Ciudad de residencia								
Arequipa	387	28,3	337	28,8	275	28,8	502	28,8
Cusco	317	23,2	263	22,5	213	22,3	401	23,0
Lima	430	31,4	371	31,7	315	32,9	506	29,0
Puno	235	17,2	198	16,9	153	16,0	335	19,2
Edad de la madre								
<18 años	28	2,0	24	2,1	19	2,0	35	2,0
18-29 años	794	58,0	674	57,7	549	57,4	1036	59,4
>30 años	547	40,0	471	40,3	388	40,6	673	38,6
Viven con pareja								
Si	1153	84,2	982	84,0	799	83,6	1482	85,0
No	216	15,8	187	16,0	157	16,4	262	15,0
Tenencia de seguro de salud								
Sí	899	65,7	768	65,7	625	65,4	1137	65,2
No	470	34,3	401	34,3	331	34,6	607	34,8
Recibió al menos un control prenatal								
Sí	1359	99,3	1162	99,4	951	99,5	1729	99,1
No	10	0,7	7	0,6	5	0,5	15	0,9
Consumo de suplemento de hierro prenatal								
Sí	1303	95,2	1114	95,3	918	96,0	1651	94,7
No	66	4,8	55	4,7	38	4,0	93	5,3
Nivel educativo de la madre								
Primaria	80	5,8	71	6,1	53	5,5	106	6,1
Secundaria	758	55,4	638	54,6	502	52,5	969	55,6
Superior	531	38,8	460	39,3	401	41,9	669	38,4
Autoidentificación étnica								
Mestizo	552	40,3	481	41,1	404	42,3	694	39,8
Quechua	697	50,9	586	50,1	475	49,7	901	51,7
Aimara	74	5,4	64	5,5	52	5,4	98	5,6
Blanco	23	1,7	20	1,7	14	1,5	27	1,6
Nativo o indígena de la Amazonia	14	1,0	11	0,9	8	0,8	15	0,9
Negro/mulato/zambo/afroperuano	4	0,3	3	0,3	2	0,2	4	0,2
Otros	5	0,4	4	0,3	1	0,1	5	0,3
Agua conectada a red pública								
Sí	1303	95,2	1113	95,2	912	95,4	1657	95,0
No	66	4,8	56	4,8	44	4,6	87	5,0
Desagüe conectado a red pública								
Sí	1266	92,5	1083	92,6	890	93,1	1605	92,0
No	103	7,5	86	7,4	66	6,9	139	8,0
Cocina a gas								
Sí	1327	96,9	1135	97,1	931	97,4	1693	97,1
No	42	3,1	34	2,9	25	2,6	51	2,9
Total	1369	100,0	1169	100,0	956	100,0	1744	100,0

G1 (Grupo 1): niños con ferritina sérica (FS) normal (>12µg/L) o ajustada (>30µg/L) en presencia de inflamación (PCR>5mg/L). G2 (Grupo 2): Niños sin inflamación (PCR ≤5mg/L), con FS normal (>12µg/L) y proteína fijadora de retinol (RBP) normal (≥0,7µmol/L). G3 (Grupo 3): Niños sin inflamación (PCR ≤5mg/L) con FS normal (>12µg/L), con receptor soluble de transferrina (RsTf) normal (≤8,3mg/L) y RBP normal (≥0,7µmol/L). G4 (Grupo 4): - Todos los niños fueron incluidos sin considerar el estado de hierro o presencia de inflamación.

Respecto al estado de hierro de los niños se encontró que, según FS, la proporción de DH en Puno fue de 29,9%, en Arequipa 22,9%, en Cusco fue 21% y en Lima 15%; mientras que aplicando RsTf, la DH alcanzó el 38,8% de niños de 6 a 8 meses de Puno, 29,7% en Cusco, 29,5% en Arequipa y 20,8% en Lima. Además, se halló que la proporción de niños con inflamación por PCR elevada en Cusco fue 18,7%, Puno 14,6%, Lima 12,3% y Arequipa 11,8% (Tabla 2).

**Tabla 2:** Deficiencia de hierro, déficit de retinol sérico e inflamación en niños de 6 a 8 meses de Lima, Arequipa, Cusco y Puno

Características	Lima		Arequipa		Cusco		Puno	
	n	%	n	%	n	%	n	%
Déficit de hierro								
Según ferritina sérica ajustada por PCR ^a^	76	15,0	115	22,9	84	21,0	100	29,9
Según ferritina sérica ajustada por AGP ^b^	69	13,6	115	22,9	81	20,2	92	27,5
Según receptor soluble de transferrina	105	20,8	148	29,5	119	29,7	130	38,8
Déficit de retinol sérico	70	13,8	72	14,3	72	18,0	63	18,8
Inflamación								
Según PCR ^a^	62	12,3	59	11,8	75	18,7	49	14,6
Según AGP ^b^	65	12,9	63	12,6	54	13,5	26	7,8

a Proteína C reactiva; ^b^ α-1-glicoproteína ácida

En la Tabla 3, se analizaron los valores promedio de los biomarcadores de hierro según grupo de estudio y ciudad, y se aprecia que el grupo 4 mostró los valores de FS y HCT significativamente más bajos, así como, el valor más alto del RsTf (p<0,05); mientras que el grupo 3 mostró el mayor valor de HCT y el más bajo de RsTf (p<0,05). Los valores de PCR en los grupos 2 y 3 fueron iguales y mostraron diferencias significativas con los grupos 1 y 4 (p<0,05), mientras que el AGP solo mostró diferencia significativa entre el grupo 3 y el resto de los grupos (p<0,05). Además, según ciudades se encontró que los valores más altos de Hb (sin corrección de altura) estaban en Puno y mostraba diferencias significativas con Lima (p<0,05); sin embargo, al observar los valores de FS y HCT los valores más altos se encontraban en Lima con diferencias significativas con las otras tres ciudades (p<0,05). El promedio de PCR más alto se encontró en Cusco y fue significativamente diferente a las otras tres ciudades (p<0,05).

**Tabla 3:** Mediciones de los biomarcadores evaluados en los niños de 6 a 8 meses incluidos en el estudio, según grupo y ciudad.

Características	n	Hemoglobina (g/dL)		Ferritina sérica (μg/L)	Receptor soluble de transferrina (mg/L)	Hierro corporal total (mg/kg)	Retinol sérico (μmol/L)	PCR (mg/L)	AGP (g/L)
		Media ± DE	Percentil 5% - 95%	Media ± DE	Media ± DE	Media ± DE	Media ± DE	Media ± DE	Media ± DE
Grupos									
G1	1369	12,2 ±1,4	9,8 - 14,5	52,0±37,1^A^	7,1 ±1,8^A^	4,4 ± 3,4^A^	1,0 ±0,3^A^	3,8 ±10,4^A^	0,6 ±0,4^A^
G2	1169	12,2 ±1,4	9,8 - 14,5	51,0 ±36,9^B^	7,1 ±1,8^B,C^	4,3 ± 3,4^B,C^	1,0 ±0,3^A,B^	2,3 ±7,3^A^	0,6 ±0,3
G3	956	12,2 ±1,4	9,9 - 14,5	52,4 ±36,7^C^	6,5 ±1,1^A,B,D^	4,7 ± 3,3^A,B,D^	1,0 ±0,3^A,C^	2,3 ±7,3^A,B^	0,6 ±0,3^A,B^
G4	1744	12,1 ±1,5	9,7 - 14,4	42,7 ±37,4^A,B,C^	7,8 ±2,6^A,C,D^	3,2 ± 4,2^A,C,D^	1,0 ±0,3^B,C^	3,8 ±10,2^B^	0,6 ±0,4^B^
Ciudad									
Lima	506	10,7 ±1,1^a^	9,0 - 12,4	47,9 ±38,1^A^	7,1 ±2,3^A^	4,3 ±3,8^A^	1,00 ±0,3^A^	3,2 ±8,8^A^	0,6 ±0,4^A^
Arequipa	502	12,2 ±1,0^a^	10,6 - 13,8	44,9 ±40,6^B^	8,1 ±2,8^A^	2,6 ±4,4^A,B^	0,9 ±0,2^A^	3,1 ±9,2^B^	0,6 ±0,4^B^
Cusco	401	12,7 ±1,1^a^	10,9 - 14,5	44,1 ±37,4^A,C^	7,8 ±2,2^A^	3,5 ±3,9^A,B,C^	0,9 ±0,3	5,0 ±11,7^A,B,C^	0,6 ±0,4^C^
Puno	335	13,3 ±1,2^a^	11,2 - 15,3	30,0 ±27,6^A,B,C^	8,0 ±3,1^A^	2,0 ±4,1^A,C^	1,0 ±0,3	3,9 ±10,4^C^	0,5 ±0,3^A,B,C^

DE: desviación estandár; PCR: proteína C reativa; AGP: α-1-glicoproteína ácida; g/dL: gramos por decilitro; μg/L: microgramos por litro; μmol/L: micromoles por litro; g/L: gramos por litro.

a Existió diferencia significativa entre todas las regiones según la comparación *post-hoc* de Bonferroni para prueba Análisis de Varianza (ANOVA). Letras mayúsculas en superíndice iguales representan diferencia estadísticamente significativa en sentido vertical entre grupos del biomarcador evaluado, determinada mediante prueba *post-hoc de Dunn* con ajuste de Bonferroni para prueba de *Kruskall-Wallis.*

G1 (Grupo 1): Niños con ferritina sérica (FS) normal (>12µg/L) o ajustada (>30µg/L) en presencia de inflamación (PCR>5mg/L); G2 (Grupo 2): Niños sin inflamación (PCR ≤5mg/L), con FS normal (>12µg/L) y proteína fijadora de retinol (RBP) normal (≥0,7µmol/L); G3 (Grupo 3): Niños sin inflamación (PCR ≤5mg/L) con FS normal (>12µg/L), con receptor soluble de transferrina (RsTf) normal (≤8,3mg/L) y RBP normal (≥0,7µmol/L); y G4 (Grupo 4): Todos los niños fueron incluidos sin considerar el estado de hierro o presencia de inflamación.

Asimismo, se determinó la media y DE de Hb, de los datos individuales, por cada 100 m s. n. m. de las altitudes incluidas en el estudio (Tabla S2 del Material Suplementario) y se analizó la distribución (media e intervalos de confianza) y tendencia de Hb por altitud para los cuatro grupos de estudio, observándose un comportamiento similar en los 4 grupos con incremento de Hb a mayor altitud (Figura S2 del Material Suplementario).

De la evaluación de las funciones de la distribución de Hb, se identificó que la exponencial ofrecía un mejor desempeño según su coeficiente de determinación; las características y valores de las ecuaciones se muestran en las Tablas S3 y S4 del Material Suplementario. En el estudio se tomó como referencia la ecuación del grupo 2 (INS 2023): 10,34249 x (1,00007 ^ Alt).

Considerando que se realizó un subanálisis de los datos disponibles de un estudio cuyo objetivo era diferente al presente, se realizó una evaluación de la precisión de las estimaciones de hemoglobina para cada ciudad y grupo predeterminado a fin de garantizar la calidad de la información reportada (Tabla S5 del Material Suplementario), misma que sirvió de insumo para la identificación de las estimaciones exponenciales según piso altitudinal.

Se estimaron los valores medios de Hb de los cuatro grupos conformados empleando una regresión exponencial; asimismo, se calculó la diferencia absoluta y relativa de las estimaciones con el ajuste de CDC/OMS y del grupo 2. En esta comparación, se encontraron diferencias negativas de -0,8 a -1,7% entre los 1400 y 2600 m s. n. m.; sin embargo, por encima de 3000 a 4300 m s. n. m. se encontraron diferencias positivas que van desde 1,5 a 6,7% con tendencia ascendente por altitud (Tabla 4). En la tabla S6 del Material Suplementario se presenta el comportamiento del incremento exponencial en las estimaciones de Hb por cada aumento de 100 m s. n. m. para cada uno de los grupos evaluados.

**Tabla 4:** Estimaciones exponenciales de los valores promedio de hemoglobina de los niños de 6 a 8 meses incluidos en el estudio según grupo por altitud geográfica de residencia.

Altitud (m s. n. m. )	Hemoglobina (g/dL)					Diferencia entre G2 y CDC/OMS	
	G1 E (IC 95%)	G2 E (IC 95%)	G3 E (IC 95%)	G4 E (IC 95%)	CDC/OMS	Absoluta (g/dL)	Relativa (%)
100	10,6 (10,3-10,9)	10,4 (9,9-11,0)	10,5 (9,9-11,0)	10,4 (10,1-10,7)	11,0	0,6	5,5
1000	11,2 (11,0-11,4)	11,1 (10,7-11,5)	11,1 (10,7-11,6)	11,0 (10,8-11,2)	11,1	0,0	0,0
1100	11,3 (11,1-11,5)	11,2 (10,8-11,6)	11,2 (10,8-11,6)	11,1 (10,9-11,3)	11,2	0,0	0,0
1200	11,4 (11,2-11,6)	11,2 (10,9-11,6)	11,3 (10,9-11,7)	11,2 (11,0-11,4)	11,2	0,0	0,0
1300	11,5 (11,3-11,6)	11,3 (10,9-11,7)	11,4 (11,0-11,8)	11,2 (11,0-11,4)	11,3	0,0	0,0
1400	11,5 (11,3-11,7)	11,4 (11,0-11,8)	11,4 (11,1-11,8)	11,3 (11,1-11,5)	11,3	-0,1	-0,9
1500	11,6 (11,4-11,8)	11,5 (11,1-11,8)	11,5 (11,2-11,9)	11,4 (11,2-11,6)	11,4	-0,1	-0,9
1600	11,7 (11,5-11,8)	11,6 (11,2-11,9)	11,6 (11,3-11,9)	11,5 (11,3-11,7)	11,4	-0,2	-1,8
1700	11,8 (11,6-11,9)	11,6 (11,3-12,0)	11,7 (11,4-12,0)	11,6 (11,4-11,7)	11,5	-0,1	-0,9
1800	11,8 (11,7-12,0)	11,7 (11,4-12,0)	11,8 (11,4-12,1)	11,6 (11,5-11,8)	11,6	-0,1	-0,9
1900	11,9 (11,8-12,0)	11,8 (11,5-12,1)	11,8 (11,5-12,1)	11,7 (11,6-11,9)	11,7	-0,1	-0,9
2000	12,0 (11,8-12,1)	11,9 (11,6-12,2)	11,9 (11,6-12,2)	11,8 (11,6-11,9)	11,7	-0,2	-1,7
2100	12,1 (11,9-12,2)	12,0 (11,7-12,2)	12,0 (11,7-12,3)	11,9 (11,7-12,0)	11,8	-0,2	-1,7
2200	12,1 (12,0-12,3)	12,1 (11,8-12,3)	12,1 (11,8-12,3)	11,9 (11,8-12,1)	11,9	-0,2	-1,7
2300	12,2 (12,1-12,3)	12,1 (11,9-12,4)	12,2 (11,9-12,4)	12,0 (11,9-12,2)	12,0	-0,1	-0,8
2400	12,3 (12,2-12,4)	12,2 (12,0-12,4)	12,3 (12,0-12,5)	12,1 (12,0-12,2)	12,1	-0,1	-0,8
2500	12,4 (12,3-12,5)	12,3 (12,1-12,5)	12,3 (12,1-12,6)	12,2 (12,1-12,3)	12,2	-0,1	-0,8
2600	12,4 (12,4-12,5)	12,4 (12,2-12,6)	12,4 (12,2-12,6)	12,3 (12,2-12,4)	12,3	-0,1	-0,8
2700	12,5 (12,4-12,6)	12,5 (12,3-12,7)	12,5 (12,3-12,7)	12,4 (12,3-12,5)	12,5	0,0	0,0
2800	12,6 (12,5-12,7)	12,6 (12,4-12,7)	12,6 (12,4-12,8)	12,4 (12,3-12,5)	12,6	0,0	0,0
2900	12,7 (12,6-12,8)	12,7 (12,5-12,8)	12,7 (12,5-12,9)	12,5 (12,4-12,6)	12,7	0,0	0,0
3000	12,8 (12,7-12,9)	12,8 (12,6-12,9)	12,8 (12,6-13,0)	12,6 (12,5-12,7)	12,8	0,0	0,0
3100	12,9 (12,8-12,9)	12,8 (12,7-13,0)	12,9 (12,7-13,0)	12,7 (12,6-12,8)	13,0	0,2	1,5
3200	12,9 (12,9-13,0)	12,9 (12,8-13,1)	13,0 (12,8-13,1)	12,8 (12,7-12,9)	13,1	0,2	1,5
3300	13,0 (12,9-13,1)	13,0 (12,8-13,2)	13,0 (12,9-13,2)	12,9 (12,8-13,0)	13,3	0,3	2,3
3400	13,1 (13,0-13,2)	13,1 (12,9-13,3)	13,1 (12,9-13,3)	13,0 (12,9-13,0)	13,4	0,3	2,2
3500	13,2 (13,1-13,3)	13,2 (13,0-13,4)	13,2 (13,0-13,4)	13,0 (12,9-13,1)	13,6	0,4	2,9
3600	13,3 (13,2-13,4)	13,3 (13,1-13,5)	13,3 (13,1-13,5)	13,1 (13,0-13,2)	13,7	0,4	2,9
3700	13,4 (13,2-13,5)	13,4 (13,2-13,6)	13,4 (13,2-13,6)	13,2 (13,1-13,3)	13,9	0,5	3,6
3800	13,4 (13,3-13,6)	13,5 (13,2-13,7)	13,5 (13,2-13,8)	13,3 (13,2-13,4)	14,1	0,6	4,3
3900	13,5 (13,4-13,7)	13,6 (13,3-13,8)	13,6 (13,3-13,9)	13,4 (13,3-13,5)	14,2	0,6	4,2
4000	13,6 (13,5-13,8)	13,7 (13,4-14,0)	13,7 (13,4-14,0)	13,5 (13,3-13,6)	14,4	0,7	4,9
4100	13,7 (13,6-13,9)	13,8 (13,5-14,1)	13,8 (13,5-14,1)	13,6 (13,4-13,7)	14,6	0,8	5,5
4200	13,8 (13,6-14,0)	13,9 (13,5-14,2)	13,9 (13,5-14,2)	13,7 (13,5-13,8)	14,8	0,9	6,1
4300	13,9 (13,7-14,1)	14,0 (13,6-14,3)	14,0 (13,6-14,3)	13,8 (13,6-13,9)	15,0	1,0	6,7

CDC: Centros para el Control y Prevención de Enfermedades; OMS: organización mundial de la salud, m s. n. m.: metros sobre el nivel del mar; g/dL: gramos por decilitro, E: estimador exponencial; IC 95%: Intervalo de confianza al 95%

G1 (Grupo 1): Niños con ferritina sérica (FS) normal (>12µg/L) o ajustada (>30µg/L) en presencia de inflamación (PCR>5mg/L). G2 (Grupo 2): Niños sin inflamación (PCR ≤5mg/L), con FS normal (>12µg/L) y proteína fijadora de retinol (RBP) normal (≥0,7µmol/L). G3 (Grupo 3): Niños sin inflamación (PCR ≤5mg/L) con FS normal (>12µg/L), con receptor soluble de transferrina (RsTf) normal (≤8,3mg/L) y RBP normal (≥0,7µmol/L). G4 (Grupo 4): Todos los niños fueron incluidos sin considerar el estado de hierro o presencia de inflamación.

La Figura 1 muestra la distribución de las estimaciones exponenciales de la hemoglobina por altitud para los cuatro grupos de estudio comparados con la curva de referencia de CDC. En general, se encontró que para todos los grupos, las curvas de Hb estaban por encima de la curva CDC desde antes de los 1000 msnm hasta aproximadamente los 3000 msnm, luego por encima de este valor las curvas de los cuatro grupos estaban por debajo de la curva CDC que muestra una mayor inflexión a mayor altura. Al comparar las cuatro curvas y la de CDC en un solo gráfico (Figura S3 del Material Suplementario), se aprecia que las curvas de los grupos 2 y 3 se superponen; la curva del grupo 1 empieza por encima del grupo 2 y luego aproximadamente a 3500 msnm cae por debajo del grupo 2. Por su parte la curva del grupo 4 siempre permanece por debajo de la curva del grupo 2.

**Figura 1:** Comportamiento de las estimaciones exponenciales de los valores medios de hemoglobina de los niños de 6 a 8 meses incluidos en el estudio según grupo por altitud geográfica de residencia: (a) Comparación de las estimaciones de CDC/OMS con el Grupo 1. (b) Comparación de las estimaciones de CDC/OMS con el Grupo 2. (c) Comparación de las estimaciones de CDC/OMS con el Grupo 3. (d) Comparación de las estimaciones de CDC/OMS con el Grupo 4.CDC: Centros para el Control y Prevención de Enfermedades, OMS: Organización Mundial de la Salud, msnm: metros sobre el nivel del mar.

La Figura 2A muestra el efecto de la altitud sobre la FS y el HCT, en ambos casos se observa una relación significativa inversa a la observada con la Hb; así a mayor altitud, menores concentraciones de FS y HCT (p<0,001) en ambos casos. En tanto que en la Figura 2B se observa una relación inversa entre Hb y HCT, así existe una correlación positiva significativa entre Hb y altitud (p<0,001); mientras que el HCT muestra una correlación negativa, también significativa (p<0,001). Asimismo, encontramos comportamientos consistentes de relación inversa entre RsTf con HCT y Hb con FS (Figura S4 y S5 del Material Suplementario).

**Figura 2A:** Correlación entre la distribución de ferritina sérica y hierro corporal total por altitud en niños peruanos de 6 a 8 meses sin inflamación [Grupo 2 - Niños sin inflamación, PCR > 5 mg/L con ferritina sérica >12 μg/L y proteína fijadora de retinol (RBP) ≥ 0,7 μmol/L]. Figura 2B. Correlación entre la distribución de hemoglobina y hierro corporal total por altitud en niños peruanos de 6 a 8 meses sin inflamación [Grupo 2 - Niños sin inflamación, PCR > 5 mg/L con ferritina sérica >12 μg/L y proteína fijadora de retinol (RBP) ≥ 0,7 μmol/L]. msnm: metros sobre el nivel del mar.

## DISCUSIÓN

El propósito del estudio fue caracterizar el estado del hierro y proponer factores de ajuste de hemoglobina por altitud. Respecto al primer punto, se encontraron mayores proporciones de DH en los niños de las ciudades con mayor altitud (Cusco y Puno), además, tenían menores valores promedio de FS, HCT y valores más altos de RsTf evidenciando menores reservas de hierro en la altura. Al analizar la hemoglobina por altitud, los resultados muestran un aumento exponencial de los valores en todos los niños por encima de 1000 msnm, lo que se explicaría como una respuesta fisiológica a la hipoxia y evidenciaría la necesidad de ajustar la estimación de hemoglobina por la altitud.

En cuanto a la estimación de factores de ajuste por altitud, fue necesario definir cuatro grupos de estudio para contrastar los resultados de las estimaciones en varias situaciones. Así, el primer grupo consideró los criterios usados en EESS para el diagnóstico de DH por FS en niños con o sin inflamación, el segundo grupo (INS 2023) se consideró de referencia porque siguió las recomendaciones del estudio BRINDA ^15^. El tercer grupo además de los criterios de BRINDA excluyó niños con DH por RsTf y el cuarto grupo incluyó a todos los niños sin excluir niños con DH o inflamación para reproducir la situación que plantean los autores que proponen puntos de corte usando datos de SIEN o ENDES ^12^^-^^14^^,^^16^. Otros estudios multinacionales que buscaban verificar factores de ajuste de hemoglobina por altitud también consideraron criterios relativamente similares para definir los grupos de estudio ^10^^,^^11^.

Se seleccionó el modelo exponencial para la ecuación de ajuste de hemoglobina por altitud, porque mostraba mejores parámetros estadísticos. Este enfoque concuerda con lo reportado por Hurtado ^7^ y Cohen ^20^ en población adulta, y Dirren ^9^ y Bartolo ^14^ en niños; aunque se diferencia de otros como CDC ^8^, Ocas-Córdova ^13^, Sharma ^10^ y Kanu ^11^ que propusieron ecuaciones cuadráticas y Miao ^21^ que propuso una ecuación lineal.

En la comparación de los valores de referencia de hemoglobina entre CDC/OMS y el presente estudio, se encontraron diferencias. Así, entre 1400 y 2600 msnm, los valores del CDC/OMS fueron menores, lo que podría indicar una subestimación de anemia en este rango de altitudes, mientras que por encima de 3000 msnm los valores del CDC/OMS fueron superiores, lo que implicaría una sobreestimación de anemia a partir de los 3100 msnm. Esta tendencia de subestimación en altitudes entre 1400 y 2600 msnm y sobreestimación por encima de 3000 msnm concuerda con lo reportado por Sharma *et al.*, en preescolares y mujeres ^10^, y Kanu *et al.*, en escolares ^11^.

Otro aspecto controversial respecto a los factores de ajuste por altitud ha sido atribuir a los pobladores de ciudades de altura de la zona sur del Perú características de otras poblaciones como las del Tíbet, donde no sería necesario el ajuste por altitud ^22^. Al respecto, se han reportado diferencias en el incremento de hemoglobina en poblaciones que viven a gran altitud con diferente origen étnico, así la población que vive en la región andina tiene valores de hemoglobina más altos que la población de Asia (Tíbet) y África (Etiopía) ^23^; por lo que estos mecanismos de adaptación de diferentes poblaciones se basarían en la expresión génica, adaptaciones fisiológicas y el estado nutricional ^24^. En el caso de la población andina, además, se ha reportado una reducción en el volumen plasmático y niveles más altos de eritropoyetina ^25^^,^^26^.

En cuanto a la distribución de FS y HCT según altitud mostraron una correlación negativa significativa (p<0,001) evidenciando que la altura no influye sobre sus valores. Además, las curvas de ambos biomarcadores mostraban una tendencia opuesta al comportamiento de la hemoglobina y del RsTf que aumentaban a mayor altitud. Los hallazgos de FS, HCT más bajos y valores altos de RsTf en los niños de tres ciudades de altitud sugieren que no tendrían suficientes reservas para aumentar la hemoglobina ^27^. Este hallazgo podría explicarse porque los niños de 6 a 8 meses requieren más hierro para duplicar el volumen sanguíneo ^3^ y por las mayores necesidades de hierro debido a la adaptación de la hemoglobina a la altitud ^28^. Aunque existen mecanismos que intentan compensar el metabolismo de hierro, como la regulación de la hepcidina ^29^; también es importante recordar que cuando las dietas son pobres en hierro, como en Perú ^30^, estos mecanismos resultan insuficientes para cubrir las necesidades de hierro, por ello los niños que viven en zonas de altura tendrían una captación inadecuada de hierro y menores reservas para cubrir las mayores demandas ^31^.

Los resultados del presente estudio tienen implicancias en la salud pública. Primero, el comportamiento observado de la hemoglobina en altitud respaldaría la necesidad de ajustar las estimaciones de hemoglobina en poblaciones de altura. Segundo, los valores de ajuste de hemoglobina estimados en el presente estudio podrían sumar evidencia para contribuir a diagnosticar anemia de manera más precisa en la altura y aportarían a un manejo más efectivo de las intervenciones con hierro en niños de ciudades con mayor altitud.

Una de las fortalezas del presente estudio radica en la selección de niños sanos que cumplían sus atenciones de salud y alimentación; además, se incluyeron varios biomarcadores de hierro e inflamación lo que permitió seleccionar niños con estado de hierro normal. Otra fortaleza fue que se contó con datos de cuatro ciudades de diferente altitud para comparar diferencias en el estado de hemoglobina por altitud. Asimismo, los análisis de los biomarcadores se realizaron en un laboratorio de referencia internacional. El presente estudio también cuenta con limitaciones. Primero, los resultados solo muestran el estado basal del hierro en una cohorte de niños; sin embargo, se debe considerar que los criterios de inclusión permitieron seleccionar niños con buen estado de hierro. Segundo, las estimaciones de los biomarcadores fueron realizadas en niños de 6 a 8 meses de edad, por lo que no podrían extrapolarse directamente a otros grupos de edad. Tercero, otras limitaciones inherentes al diseño muestral fueron que no se calculó un tamaño de muestra para estimar valores de hemoglobina por cada 100 metros de altitud, sino por cada ciudad seleccionada; esto podría afectar las estimaciones de hemoglobina. Al respecto, calculamos la precisión de las estimaciones encontrando valores aceptables. Además, la muestra del estudio fue recolectada de forma no probabilística lo que podría afectar la validez externa de los hallazgos; empero, se debe considerar que la muestra procede de una cohorte de base poblacional de niños atendidos en EESS del MINSA y que los estudios para elaborar patrones de referencia enfatizan más estrategias muestrales para garantizar poblaciones sanas que poblaciones representativas ^32^. Asimismo, se incluyeron cuatro ciudades ubicadas a diferentes altitudes, por lo que la representatividad geográfica podría ser limitada; sin embargo, diversos estudios con datos de todo el país ^12^^-^^14^ muestran que la altitud es la variable más influyente para el comportamiento de la hemoglobina. Una limitación adicional fue que en el presente estudio no se pudo determinar los niveles de hepcidina en los participantes del estudio; no obstante, para los objetivos del presente estudio el grupo BRINDA ^15^ recomienda determinar solo los biomarcadores incluidos en el estudio.

En conclusión, el estudio encontró mayores proporciones de DH en niños de ciudades de altura como Cusco y Puno, y que sus valores de reserva de hierro son más bajos comparados con Lima. Además, la Hb mostró una distribución exponencial según altitud, evidenciando la necesidad de aplicar un factor de ajuste y en base al modelo exponencial se propuso una ecuación de ajuste para Hb en niños que residen en ciudades de altura. En la comparación de los valores estimados por CDC/OMS y el presente estudio, los datos sugieren una subestimación de anemia entre 1400-2600 msnm y una sobreestimación entre 3000-4300 msnm. Se necesitan estudios más amplios en diferentes grupos etarios y fisiológicos que viven a gran altura y en varias regiones para corroborar estos hallazgos; además, deben incluir información sobre la dieta ingerida y el balance metabólico del hierro.
